# Longitudinal and transversal resonant tunneling of interacting bosons in a two-dimensional Josephson junction

**DOI:** 10.1038/s41598-021-04312-6

**Published:** 2022-01-12

**Authors:** Anal Bhowmik, Ofir E. Alon

**Affiliations:** 1 Department of Mathematics, University of Haifa, 3498838 Haifa, Israel; 2 Haifa Research Center for Theoretical Physics and Astrophysics, University of Haifa, 3498838 Haifa, Israel

**Keywords:** Physics, Ultracold gases

## Abstract

We unravel the out-of-equilibrium quantum dynamics of a few interacting bosonic clouds in a two-dimensional asymmetric double-well potential at the resonant tunneling scenario. At the single-particle level of resonant tunneling, particles tunnel under the barrier from, typically, the ground-state in the left well to an excited state in the right well, i.e., states of different shapes and properties are coupled when their one-particle energies coincide. In two spatial dimensions, two types of resonant tunneling processes are possible, to which we refer to as longitudinal and transversal resonant tunneling. Longitudinal resonant tunneling implies that the state in the right well is longitudinally-excited with respect to the state in the left well, whereas transversal resonant tunneling implies that the former is transversely-excited with respect to the latter. We show that interaction between bosons makes resonant tunneling phenomena in two spatial dimensions profoundly rich, and analyze these phenomena in terms of the loss of coherence of the junction and development of fragmentation, and coupling between transverse and longitudinal degrees-of-freedom and excitations. To this end, a detailed analysis of the tunneling dynamics is performed by exploring the time evolution of a few physical quantities, namely, the survival probability, occupation numbers of the reduced one-particle density matrix, and the many-particle position, momentum, and angular-momentum variances. To accurately calculate these physical quantities from the time-dependent many-boson wavefunction, we apply a well-established many-body method, the multiconfigurational time-dependent Hartree for bosons (MCTDHB), which incorporates quantum correlations exhaustively. By comparing the survival probabilities and variances at the mean-field and many-body levels of theory and investigating the development of fragmentation, we identify the detailed mechanisms of many-body longitudinal and transversal resonant tunneling in two dimensional asymmetric double-wells. In particular, we find that the position and momentum variances along the transversal direction are almost negligible at the longitudinal resonant tunneling, whereas they are substantial at the transversal resonant tunneling which is caused by the combination of the density and breathing mode oscillations. We show that the width of the interparticle interaction potential does not affect the qualitative physics of resonant tunneling dynamics, both at the mean-field and many-body levels. In general, we characterize the impact of the transversal and longitudinal degrees-of-freedom in the many-boson tunneling dynamics at the resonant tunneling scenarios.

## Introduction

Tunneling is a purely quantum mechanical phenomenon which takes place in classically-forbidden region, originally intended to account for α-decay, fusion, and fission in nuclear physics^1,2^. Apart from nuclear physics, tunneling occurs naturally in photoassociation and photodissociation processes, and in solid-state structures^3–7^. Ultracold quantum gases have been the subject of research to simulate solid-state systems^8–15^. In the context of the study of tunneling dynamics of ultracold atoms, a double-well potential with static barrier is a standard example. Most of the tunneling phenomena were investigated with ultracold atoms in one dimension, either in a double-well potential or in periodic optical lattices, and focused on the atomic motion in the lowest band, i.e., particularly with the ground state^16–23^. Moreover, it was shown in a double-well potential that the inclusion of higher single-particle levels is fundamentally important for correlations^24,25^. Josephson-like dynamics of a Bose–Einstein condensate of rubidium atoms was investigated in the second Bloch band of an optical square lattice^26^. Unconventional orbital superfluidity in the *P*- and *F*-bands of a bipartite optical square lattice was achieved in^27,28^. Recently, we explored the tunneling dynamics of ultracold bosons in a symmetric double-well potential where the atomic motion lies in the ground as well as excited bands^29^. Dynamics and quantum optimal control of different complex atomic quantum systems and the transition from the ground to complicated excited states have been investigated in anharmonic trap or in optical lattices^30–32^. Moreover, complicated tunneling dynamics of ultracold atoms, such as, Fermi-Fermi mixture^33,34^ and bosonic impurities with a bosonic medium^35,36^ were studied in a double-well potential.

The interband quantum tunneling of ultracold atoms between the ground and excited bands can occur by breaking the symmetry of the two wells of a symmetric double-well potential, say, by generating an asymmetric double-well potential. This asymmetric double-well potential can be understood as a unit cell of a tilted optical lattice^37–40^. Also, an external electromagnetic field can induce interband transitions as demonstrated in the spectroscopy of Wannier-Stark levels^41^. Here, we are interested in the interband quantum tunneling in an asymmetric double-well potential, typically describes in the tilted optical lattice. Per definition, the interband tunneling in an asymmetric double-well potential occurs when the energy of the ground state on one side of the double-well coincides with the energy of an excited state on the other side. This leads to tunneling between those states which is resonantly enhanced by the energy matching, usually referred to as resonantly enhanced tunneling^42,43^.

Resonantly enhanced tunneling phenomenon has been observed in various fields of research, such as in a nonlinear effect in a Mott insulator by creating a particle-hole excitation^44^, in the presence of many-body coherences^45^, in waveguide arrays^46^, in an optical lattice with an external magnetic field producing a Zeeman splitting of the energy levels^47^, in the two-terminal current-voltage characteristics of a finite superlattice^48^, in solid states systems such as superlattice^5,49^, and in accelerated optical lattice potentials^38,39^.

Hitherto, resonantly enhanced tunneling is studied when the resonant condition of energy matching of the two wells is along the direction of the barrier^42,43,50^. We name it longitudinal resonant tunneling. In Ref.^43^, the condensates were prepared in a one-dimensional optical lattice with an additional Stark force and determined the tunneling rate by the Landau-Zener formula^51–55^. Zenesini *et al. * experimentally investigated the impact of atom-atom interactions on the resonantly enhanced tunneling process and presented a complementary theoretical description of Landau-Zener tunneling for ultracold atoms in periodic potentials^42^. The out-of-equilibrium quantum mean-field and many-body dynamics of interacting bosons were recently explored in a one-dimensional asymmetric double-well potential^50^ using the multi-configurational time-dependent Hartree for bosons (MCTDHB) method^56–58^. Precisely, the interacting bosons were prepared in the ground state of the left well (harmonic potential) and the mechanism of the tunneling process in the resulting double-well was studied by analyzing the time-evolution of the survival probability, depletion and fragmentation, and the many-particle position and momentum variances^50^.

Resonant tunneling in a two-dimensional (2D) double-well set-up brings interesting new questions, especially, on the role of transverse excitations. Also, in 2D double-well, there can be a resonant tunneling phenomenon which does not exist in one-dimension, namely, transversal resonant tunneling when the energy-matching condition of the two wells of an asymmetric double-well is satisfied along the transverse direction of the barrier. In this work, we focus on the longitudinal and transversal resonant tunneling for the initial bosonic structures of the ground and excited states in a 2D asymmetric double-well potential. Here the bosons are loaded in the left well of the double-well potential and the barrier is formed along the *x*-direction. In order to investigate the tunneling dynamics in the longitudinal resonant scenario, we consider two different structures of bosonic clouds, i.e., the ground and transversely excited (*y*-excited) states. Although, the ground state has a one-dimensional analog, but to create a transversely excited state, one requires at least a 2D geometry^29^. Therefore, to investigate the effect of the longitudinal resonant tunneling on the very basic state which includes transverse excitations is of fundamental interest. Moreover, we investigate the impact of the transverse direction on the tunneling dynamics of the ground state at the longitudinal resonant tunneling condition. In the transversal resonant scenario, the bosonic structures are assumed to be the ground and longitudinally excited (*x*-excited) states. These two states have one-dimensional analogs but the transversal resonant tunneling does exist only in a 2D geometry. Therefore, the role of longitudinal excitations in transversal resonant tunneling can be examined. In order to accurately explore the out-of-equilibrium tunneling dynamics for both resonant tunneling scenarios, we solve the underlying time-dependent many-boson Schrödinger equation using the MCTDHB method^57,58^. We solve the quantum dynamics of all the bosonic clouds at the mean-field and many-body levels.

Tunneling dynamics for all the bosonic structures are analyzed by the time evolution of various physical quantities, namely, the survival probability, loss of coherence, depletion and fragmentation, and the many-particle position, momentum, and angular-momentum variances. Even when the bosons are fully condensed, the variance is a sensitive probe of correlations^59^. Therefore, to display the effects of the quantum correlations on the variances of different operators, we compare the mean-field and many-body variances in addition to the corresponding comparison of the survival probabilities. We notice that the rate of growth of the quantum correlations depends on the shape of the bosonic clouds, presence of transverse excitations in the system, and the resonant tunneling condition. The interconnection between the density oscillations and the variances are discussed both at the longitudinal and transversal resonant tunneling scenarios. We show that correlations have different consequences on the various quantities discussed in this work. In general, we ask how the transverse degrees-of-freedom, perpendicular to the junction, can influence the time evolution of various physical quantities in a 2D asymmetric double-well potential at the resonant tunneling scenario. We find that the time evolutions of the variances behave completely differently in the longitudinal and transversal resonant tunneling conditions.

## Theoretical method

The dynamics of *N* interacting bosons in a two-dimensional trap can be described by the time-dependent many-body Shrödinger equation,

1 $$\begin{aligned} \hat{H}\Psi = i\dfrac{\partial \Psi }{\partial t},  \hat{H}({\mathbf{r}} _1. {\mathbf{r}} _2,\ldots , {\mathbf{r}} _N)=\sum _{j=1}^{N} [\hat{T}({\mathbf{r}} _j)+\hat{V}({\mathbf{r}} _j)]+\sum _{j<k} \hat{W}({\mathbf{r}} _j-{\mathbf{r}} _k), \end{aligned}$$

where $\hat{T}({\mathbf{r}} )$ and $\hat{V}({\mathbf{r}} )$ represent the kinetic and potential energy terms, respectively. The interaction between the bosons is repulsive and considered as a Gaussian function^60^ $W({\mathbf{r}} _1-{\mathbf{r}} _1)=\lambda _0\dfrac{e^{-({\mathbf{r}} _1-{\mathbf{r}} _2)^2/2\sigma ^2}}{2\pi \sigma ^2}$ with $\sigma =0.25\sqrt{\pi }$. In Appendix B, we investigate the role of the width of the interparticle interaction potential and demonstrate explicitly that the mean-field and many-body physics found in this work do not qualitatively depend on this width. Here, $\lambda _0$ is the interaction strength and it is related with the interaction parameter $\Lambda$ by $\Lambda =\lambda _0(N-1)$ where *N* is the number of bosons. Note that the stronger the interaction parameter $\Lambda$ is, the larger the many-body effects. Here the model inter-bosons interaction does not have any qualitative impact on the physics described here.

In order to solve Eq. (1) in-principle numerically exactly, we use the MCTDHB method^20,21,50,56,57,59,61–70,70–79^. MCTDHB incorporates a variational optimal ansatz which is generated by distributing the *N* bosons over *M* time-dependent orbitals. The MCTDHB wavefunction is described as^57^

2 $$\begin{aligned} |\Psi (t)\rangle =\sum _{\{\mathbf{n}\}}C_{\mathbf{n}}(t)|\mathbf{n };t\rangle , \end{aligned}$$

where $C_n(t)$ is the expansion coefficients and $|{\mathbf{n}} ;t\rangle = |n_1, n_2,\ldots , n_M;t\rangle$. The number of time-dependent permanents $|{\mathbf{n}} ;t\rangle$ is $$\big ({\begin{matrix} N+M-1\\ N \end{matrix}}\big )$$. $M= 1$ in the set of permanents, $|{\mathbf{n}} ;t\rangle$, of Eq. (2) represents the Gross-Pitaevskii ansatz and solves the time-dependent Gross-Pitaevskii equation^80^. The accuracy of the wavefunction increases with *M* and achieves the convergence of different physical quantities of interest discussed in this work, such as the survival probability, depletion and fragmentation, and the many-particle position, momentum, and angular-momentum variances. The numerical implementation of the real-time dynamics employed in this work can be found in^81,82^.

## Results and discussion

Here, we divide the section into two parts, namely, longitudinal and transversal resonant tunneling. In each part, we investigate the time evolution of various physical quantities, such as the survival probability, loss of coherence, and the variances of the position, momentum, and angular-momentum many-particle operators when the symmetry of the double-well potential is broken and it reaches to the resonant tunneling condition. The quantities discussed here incorporate a detailed information of the time-dependent many-boson wave function, explicitly, the density, reduced one-particle density matrix, and reduced two-particle density matrix.

### Longitudinal resonant tunneling

**Figure 1:**
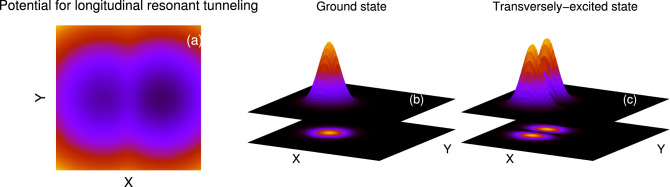
(**a**) Schematic diagrams for the asymmetric 2D double-well potential for longitudinal resonant tunneling, described in Eq. (4). Here the the right well is lower than the left well. Panels (**b**) and (**c**) show the initial density distributions for the ground and transversely-excited states, respectively.

We investigate the dynamics of a few bosonic clouds prepared as either the ground or transversely-excited state for the longitudinal resonant tunneling phenomena in asymmetric 2D double-well potential. In the longitudinal resonant tunneling, we consider that the bosons are initially prepared in the left well,

3 $$\begin{aligned} V_L(x,y)=\dfrac{1}{2}(x+2)^2+\dfrac{1}{2}y^2-cx, \end{aligned}$$

of a double-well potential, where *c* is the asymmetry parameter. The bosons are taken in the state of either as the non-interacting ground, $\Psi _G=\dfrac{1}{\sqrt{\pi }}F(x,y)$, or as the transversely-excited, $\Psi _Y=\sqrt{\dfrac{2}{\pi }}y F(x,y)$, states, where $F(x,y)=exp[-\{(x+2)^2+y^2\}/2]$. Figure 1 shows the initial density distributions of $\Psi _G$ and $\Psi _Y$. To explore the dynamics of the bosonic clouds, we suddenly quench the inter-particle interaction at $t=0$ from $\Lambda =0$ to $\Lambda =0.01\pi$ and simultaneously change the trapping potential from $V_L(x,y)$ to the longitudinally-asymmetric 2D double-well potential, $V_T(x,y)$. Here the asymmetry implies that the right well is lower than the left well. This is achieved by adding a linear term in the longitudinal direction. The mathematical form of $V_T(x,y)$ is (see Fig. 1a):

4 $$\begin{aligned} V_T(x,y)= {\left\{ \begin{array}{ll} \dfrac{1}{2}(x+2)^2+\dfrac{1}{2}y^2-cx,  x<-\dfrac{1}{2}, -\infty< y<\infty , \\ \dfrac{3}{2}(1-x^2)+\dfrac{1}{2}y^2-cx,  |x|\le \dfrac{1}{2}, -\infty< y<\infty , \\ \dfrac{1}{2}(x-2)^2+\dfrac{1}{2}y^2-cx,  x>+\dfrac{1}{2} -\infty< y<\infty . \end{array}\right. } \end{aligned}$$

To accurately tackle the many-body physics involved in the two-dimensional bosonic Josephson junctions, we have performed the MCTDHB computations with different time-dependent orbitals for different initial bosonic structures. For $\Psi _G$, the many-body computation is performed with $M=6$ time-dependent orbitals, while for $\Psi _Y$ with $M=10$ time-dependent orbitals. The convergence of the quantities, discussed in this work, with the orbital numbers are shown in the supplemental materials. For the numerical solution of the longitudinal resonant tunnelling, the considered box size is $[-10, 10)\times [-10, 10)$ with periodic boundary conditions having a grid of $64\times 64$ points. Convergence of the results with respect to the number of grid points has been checked and presented in the supplemental materials. Adequacy of the 2D box size for the longitudinal tunneling scenario has been checked. For the many-body dynamics, the number of bosons and interaction parameter are chosen as $N=10$ and $\Lambda =0.01\pi$. The mean-field dynamics are also computed with same interaction parameter $\Lambda$ to relate the results with the respective many-body ones. It is noted that as the considered interparticle interaction in the dynamics is weak, the preparation of the initial state in the relaxation process either with noninteracting bosons or with $\Lambda =0.01\pi$ does not affect the dynamical behavior of the properties discussed in this work. The consistency of preparation of the initial state is documented in the supplemental materials of^29^. The natural units $\hbar =m=1$ are employed throughout this work.

At first we investigate how the longitudinal resonant tunneling phenomena of $\Psi _G$ and $\Psi _Y$ are achieved by gradually increasing the asymmetry parameter, *c*, in the longitudinally-asymmetric 2D double-well potential described in Eq. (4). Further, we discuss the time-evolution of the different physical quantities, mentioned above, for $\Psi _G$ and $\Psi _Y$ at the resonant tunneling condition and compare them with the corresponding results of symmetric 2D double-well potential, which can be obtained at $c=0$ from Eq. (4). The investigation is performed and compared at the mean-field and many-body levels of theory.

In this work, $\Psi _G$ and $\Psi _Y$ are prepared in the left well of a 2D double-well potential. When we gradually increase the value of *c* starting from zero, the symmetry of the double-well potential breaks and the left well becomes the upper well and, consequently, the right well becomes the lower well. For some special values of *c*, the one-body energy of the left well coincides with one of the higher one-body energy levels of the right well, resulting in an enhanced tunneling of bosons from the left well to the right well. This enhancement of tunneling is usually referred to as the resonant tunneling in a double-well potential, see in this context^42,43^. From Eq. (4), we can find out that at any asymmetry parameter *c*, the energy difference of one-body spectrum between the two wells becomes 4*c*. Analytically, for harmonic left and right wells, one can realize that the resonant tunneling occurs when 4*c* will be equal to an integer.

**Figure 2:**
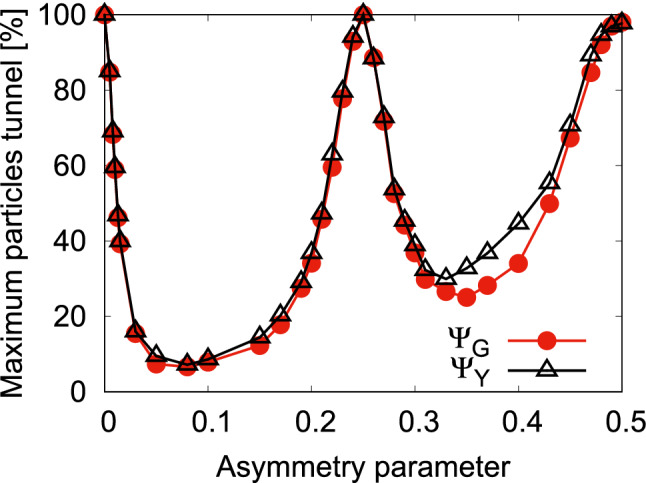
Variation of the maximal number of particles tunneling [in %] from the left to the right well with the asymmetry parameter *c* for the states $\Psi _G$ and $\Psi _Y$ at the mean-field level. The data is generated from the time-dependent solution of the Gross-Pitaeveskii equation. We show here dimensionless quantities.

Now, we show how the gradual increase of asymmetry between the two wells of a double-well potential influences the tunneling of particles. In Fig. 2, we present, at the mean-field level, the variation of the maximal number of particles tunneling from the left to the right well for the states $\Psi _G$ and $\Psi _Y$ with the asymmetry parameter *c*. This maximal number of particles in the right well is determined from $\int \limits _{x=0}^{+\infty }\int \limits _{y=-\infty }^{+\infty }dx dy \dfrac{\rho (x,y;t)}{N}$, where $\rho (x,y;t)$ is the density of the bosonic cloud, at a particular instant of time when the right well is maximally occupied by the particles. Here we start from the symmetric double-well potential. At $c=0$, when the bosons are allowed to evolve in time, we observe that 100% of the bosons can tunnel from the left to right well, signifying the delocalization of the one-particle eigen-functions in a symmetric double-well. Now, a small increase of asymmetry between the wells makes the one-particle state to be only partially delocalized over both wells, leading to a suppression of tunneling of particles. Further increase of the asymmetry, beyond $c=0.1$, the one-particle state slowly begins to be delocalized in one of the wells, and the tunneling of particles increases. At $c=0.25$, we find well-delocalization of the one-particle state in one of the well which leads to a complete tunneling of particles or specifically, refer to as resonant tunneling condition, when one-body energy of the left well matches with the energy of one of the one-body excited states of the right well. Similarly, the second resonant tunneling appears at $c=0.5$. As the system is weakly interacting, we find that the maximum tunneling of particles is 100% both for $\Psi _G$ and $\Psi _Y$ at $c=0$, $c=0.25$, and $c=0.5$. Also, we observe a small difference of maximum tunneling of particles between the results of $\Psi _G$ and $\Psi _Y$ at off-resonant tunneling conditions. Here, it is noted that if the bosons are prepared in the right well (lower well), the gradual increase of asymmetry between the wells reduces the maximal number of bosons tunneling to the left well. Further increase of asymmetry between the wells, at $c\gtrsim 0.1$, the bosons become trapped in the right well, and produces only a mild breathing motion, mainly due to the quenching of the interaction.

Next, we focus on both the resonant tunneling values of *c* and study how the many-body correlations affects the dynamical behaviors of the survival probability, loss of coherence, and the many-particle position, momentum, and angular-momentum variances. Moreover, we will display the corresponding analysis of the results obtained for the symmetric double-well potential to serve as a reference. In addition, we will present a comparison study of the mean-field and many-body results both for $\Psi _G$ and $\Psi _Y$.

**Figure 3:**
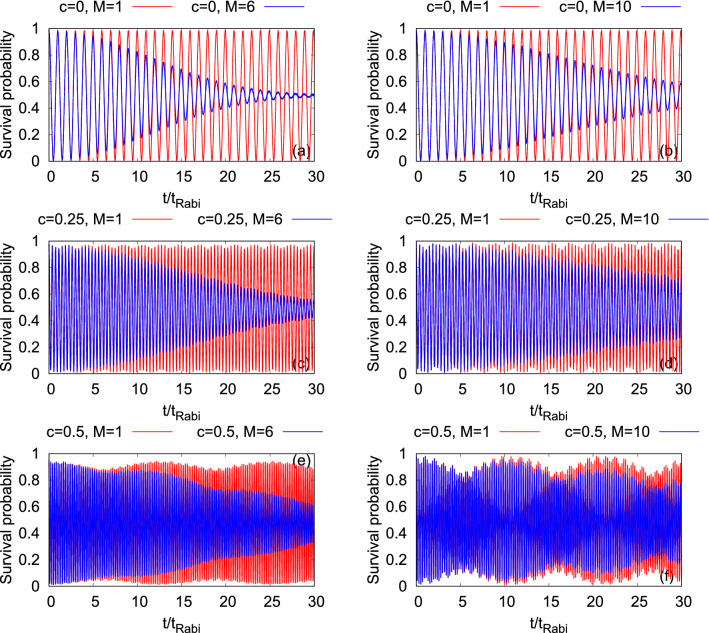
Dynamics of the survival probability of $\Psi _G$ (left column) and $\Psi _Y$ (right column) in the left well of longitudinally-asymmetric 2D double-well potential of $N=10$ bosons with the interaction parameter $\Lambda =0.01\pi$. The first row presents the results for the symmetric double-well potential. Second and third rows show the survival probabilities at the resonant tunneling with asymmetry parameter, $c=0.25$ and 0.5, respectively. $M=1$ signifies the mean-field results. The many-body dynamics are computed with $M= 6$ time-dependent orbitals for $\Psi _G$ and $M= 10$ time-dependent orbitals for $\Psi _Y$. We show here dimensionless quantities. Color codes are explained in each panel.

In order to adequately capture the time evolutions of $\Psi _G$ and $\Psi _Y$ between the two wells of a double-well potential, we examine the survival probability in the left well, $P_L(t)=\int \limits _{x=-\infty }^{0}\int \limits _{y=-\infty }^{+\infty }dx dy \dfrac{\rho (x,y;t)}{N}$, where $\rho (x,y;t)$ is the density of the bosonic cloud. We compare between the results of the mean-field and many-body dynamics of $P_L(t)$ in Fig. 3 for the asymmetry parameters, $c=0$, 0.25, and 0.5. Here we want to mention that, to have a proper comparison among all the results presented in this paper, the time-scale for the dynamics is set to be equal to the Rabi oscillations $(t_{Rabi})$ of the symmetric double-well trap (when $c=0$). We find that $t_{Rabi} = 132.498$ for the symmetric 2D double-well potential^29^.

In Fig. 3, we observe, for both $\Psi _G$ and $\Psi _Y$, back and forth of the density between the left and right wells with the essentially same frequency of oscillations at a particular value of *c*. In the non-interacting system, at $t=0$, $\Psi _G$ and $\Psi _Y$ lie approximately on the lowest band along the direction of the barrier and when there is an interaction quench, more bands are coupled. Note that we use the notion of (excited) bands interchangeable with (excited) states although we have a two-site trap. Both the frequency and amplitude of the tunneling oscillations of $\Psi _G$ are essentially similar to those of $\Psi _Y$ at the mean-field level. But at the many-body level, only the frequency of the tunneling oscillations remains very similar for $\Psi _G$ and $\Psi _Y$ at a particular value of *c*, while the amplitudes show a substantial difference as time progresses. The difference in the many-body tunneling amplitude between $\Psi _G$ and $\Psi _Y$ occurs as more states start to couple with time to $\Psi _Y$ in comparison to $\Psi _G$, see Fig. S3 in the supplemental materials. At $c=0.25$ and 0.5, the ground state energy of the left well matches with energies of the second (which is two-fold degenerate, $x\Psi _G$ and $y\Psi _G$) and third (which is three-fold degenerate, $xy\Psi _G$, $(x^2-1)\Psi _G$, and $(y^2-1)\Psi _G$) excited states of the right well, respectively. But, in the process of tunneling, $\Psi _G$ couples to $x\Psi _G$ and $(x^2-1)\Psi _G$ of the right well at $c=0.25$ and 0.5, respectively. Similarly, $\Psi _Y$ couples respectively to $x\Psi _Y$ and $(x^2-1)\Psi _Y$ of the right well. The transitions, $\Psi _G\rightarrow x\Psi _G$, $\Psi _G\rightarrow (x^2-1)\Psi _G$, $\Psi _Y\rightarrow x\Psi _Y$, and $\Psi _Y\rightarrow (x^2-1)\Psi _Y$, are allowed because of symmetry $y\rightarrow -y$. As $\Psi _G$ and $\Psi _Y$ mainly couple to those excited states which have excitations only along the *x*-direction, we call this tunneling the longitudinal resonant tunneling.

For both $\Psi _G$ and $\Psi _Y$, the short-time mean-field and many-body dynamics of $P_L(t)$ overlap for the interaction strength taken here. This condition imitates the so-called infinite-particle limit of the time-dependent many-boson Schrödinger equation^83,84^. Contrary to the mean-field dynamics, one can find a signature of the quantum correlations in the many-body dynamics of $P_L(t)$ in terms of incomplete tunneling of the densities and, consequently, the amplitude of the oscillations of $P_L(t)$ gradually decays. The decay in the amplitude of the many-body $P_L(t)$ signifies a collapse in the density oscillations, which is more pronounced for $\Psi _G$ than for $\Psi _Y$. However, as *c* increases, the decay rates of $\Psi _G$ and $\Psi _Y$ become smaller. For a certain value of *c*, the decay rate of $\Psi _G$ is larger compared to the corresponding rate of $\Psi _Y$, suggesting that in the tunneling process, the many-body effects develops in a different rate for different initial structures of the bosonic clouds. Explicitly, we observe that having transverse excitation delays the many-body process of the density oscillations collapse. Moreover, at $c=0.5$, the time evolution of $P_L(t)$ exhibits a partial revival for both the states.

**Figure 4:**
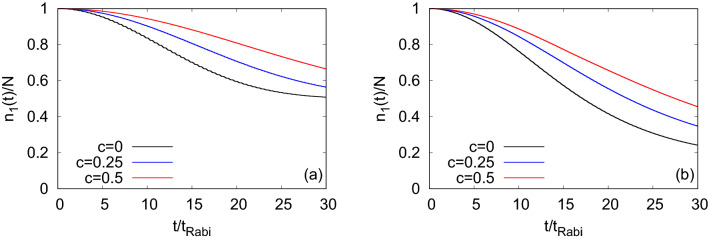
Time-dependent occupation per particle of the first natural orbital, $\dfrac{n_1(t)}{N}$, in a longitudinally-asymmetric 2D double-well potential for $N=10$ bosons and interaction parameter $\Lambda =0.01\pi$. Panels (**a**) and (**b**) are for $\Psi _G$ and $\Psi _Y$, respectively. The asymmetry parameters are $c=0$, 0.25, and 0.5. The data have been obtained with $M= 6$ time-dependent orbitals for $\Psi _G$ and $M= 10$ time-dependent orbitals for $\Psi _Y$. We show here dimensionless quantities. Color codes are explained in each panel.

Signature of a growing degree of quantum correlations is already found in terms of decay in the amplitude of the time evolution of many-body $P_L(t)$. Now, we will discuss how this gradual increase of the many-body correlations can affect the coherence of the condensates, i.e., bosonic clouds of $\Psi _G$ and $\Psi _Y$, when the resonant tunneling occurs. To compare the results, we also show the loss of coherence for the tunneling of the above considered bosonic clouds in the symmetric double-well potential as a reference. In Fig. 4, we present the time evolution of the condensate fraction, $\dfrac{n_1(t)}{N}$, obtained by diagonalizing the reduced one-particle density matrix of the time-dependent many-boson wave-function (Eq. 2)^85,86^. The general feature of the occupation of the first natural orbital is found to be decreasing with time having a weak oscillatory background for all values of asymmetry parameters. It is observed that $\Psi _Y$ loses coherence faster than $\Psi _G$ for a particular value of *c*, suggesting that inclusion of the transverse excitation enhances fragmentation in $\Psi _Y$^29^. We find that the rate of loss of coherence becomes slower when one move from $c=0$ to the first resonant tunneling value at $c=0.25$ and further slower at the second resonant tunneling.

In the context of occupations of the first natural orbital, it is worthwhile to mention the occupancy of higher natural orbitals. We find that, as the time passes by and fragmentation of the condensate grows, the occupancy of all higher natural orbitals gradually increases. The occupancy of higher natural orbitals have direct impact on the different many-body quantities discussed later, i.e, on the mechanism of many-body resonant tunneling in 2D double-wells. The detailed process of fragmentation with their convergences are discussed in the supplemental materials.

Having explicated the development of many-body correlations and their effects on the time evolution of the survival probability and coherence of the bosonic clouds, we find further information of the time-dependent many-particle wavefunction and, especially, the signature of the depletion and fragmentation on the condensate. As the variance is a sensitive probe of correlation^59^, we graphically analyze the dynamical behavior of the variances of a few fundamental quantum mechanical observables which are mostly influenced by the longitudinal resonant tunneling. These are the position and momentum operators along the *x*-direction, and the angular-momentum operator.

**Figure 5:**
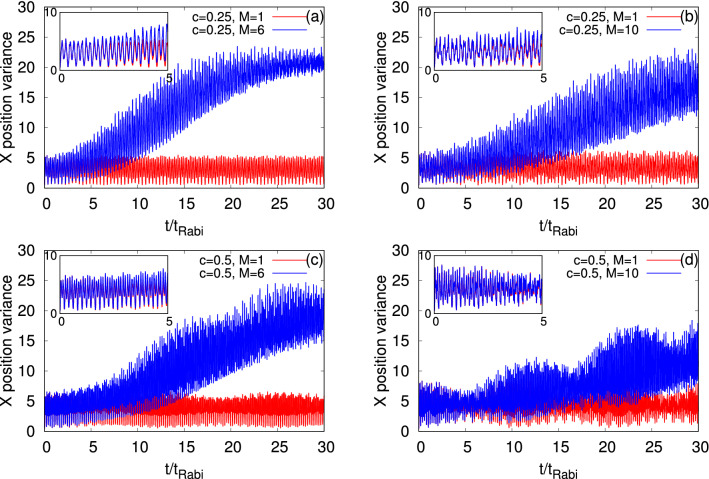
Time-dependent position variance per particle along the *x*-direction, $\dfrac{1}{N}\Delta _{{\hat{X}}}^2(t)$, in a longitudinally-asymmetric 2D double-well potential for $\Psi _G$ (left column) and $\Psi _Y$ (right column) of $N=10$ bosons with the interaction parameter $\Lambda =0.01\pi$. The upper and lower rows show $\dfrac{1}{N}\Delta _{{\hat{X}}}^2(t)$ at the resonant tunneling with asymmetry parameter, $c=0.25$ and 0.5, respectively. $M=1$ signifies the mean-field results. The many-body dynamics are computed with $M= 6$ time-dependent orbitals for $\Psi _G$ and $M= 10$ time-dependent orbitals for $\Psi _Y$. From $t=0$ to 5$t_{Rabi}$ timescale of $\dfrac{1}{N}\Delta _{{\hat{X}}}^2(t)$ is highlighted in the inset of each panel. We show here dimensionless quantities. Color codes are explained in each panel.

Figure 5 displays the time evolution of the many-particle position variance per particle along the *x*-direction, $\dfrac{1}{N}\Delta _{\hat{X}}^2(t)$, in the longitudinally-asymmetric 2D double-well potential with asymmetry parameters, $c=0.25$ and 0.5 for the bosonic clouds of $\Psi _G$ and $\Psi _Y$ (see Appendix A for the mathematical form of the many-particle variance and its basic properties). The mean-field and many-body $\dfrac{1}{N}\Delta _{\hat{X}}^2(t)$ for the symmetric 2D double-well potential for $\Psi _G$ and $\Psi _Y$ are demonstrated in Fig. S1 of the supplemental materials. As a general feature, we find that the many-body and mean-field values of $\dfrac{1}{N}\Delta _{\hat{X}}^2(t)$ are oscillatory in nature. Due to the correlations, the average value of the many-body $\dfrac{1}{N}\Delta _{\hat{X}}^2(t)$ deviates—it rather significantly increases—from the corresponding mean-field results for all asymmetry parameters and bosonic clouds. Although, we found in Fig. 4 that $\Psi _Y$ is always more fragmented than $\Psi _G$, the deviations in the values of $\dfrac{1}{N}\Delta _{\hat{X}}^2(t)$ are always smaller for $\Psi _Y$ compared to $\Psi _G$ at a particular value of *c*, apart for short-times at $c=0.5$. Therefore, one can say that the many-body dynamics is complicated and there is no one-to-one correlation between the different many-body properties in the junction, such as fragmentation and $\dfrac{1}{N}\Delta _{\hat{X}}^2(t)$, but it suggests how the transverse excitations participating in the dynamics can impact the fluctuations in measuring an observable. Unlike the mean-field dynamics, the many-body $\dfrac{1}{N}\Delta _{\hat{X}}^2(t)$ oscillates with a growing amplitude until it reaches an equilibrium value which is more evident at $c=0$ for both the states (see Fig. S1 of the supplemental materials) and at $c=0.25$ for $\Psi _G$. This equilibrium value of $\dfrac{1}{N}\Delta _{\hat{X}}^2(t)$ is reached when the density oscillations collapse as observed in the time evolutions of $P_L(t)$ in Fig. 3. At $c=0.5$, we notice that the amplitude of the oscillations of $\dfrac{1}{N}\Delta _{\hat{X}}^2(t)$ are also oscillatory, more pronouncedly for $\Psi _Y$, which is consistent with the density oscillations. In the dynamics of $\dfrac{1}{N}\Delta _{\hat{X}}^2(t)$, we observe two kinds of oscillations, i.e., small frequency with large amplitude and high frequency with small amplitude oscillations. The former one comes from the density oscillations and the latter one occurs due to the breathing-mode oscillations of the system. At the resonant values of *c*, the high frequency with small amplitude oscillations are more clearly visible.

**Figure 6:**
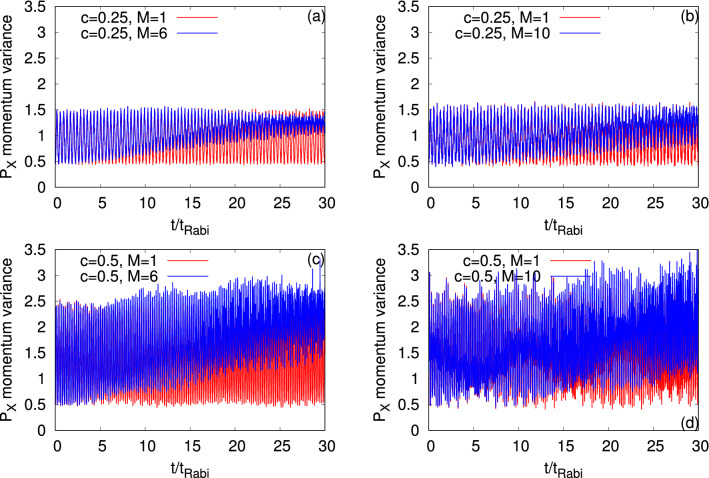
Time-dependent momentum variance per particle along the *x*-direction, $\dfrac{1}{N}\Delta _{{\hat{P}_X}}^2(t)$, in a longitudinally-asymmetric 2D double-well potential for $\Psi _G$ (left column) and $\Psi _Y$ (right column) of $N=10$ bosons with the interaction parameter $\Lambda =0.01\pi$. The upper and lower rows show $\dfrac{1}{N}\Delta _{{\hat{P}_X}}^2(t)$ at the resonant tunneling with asymmetry parameter, $c=0.25$, and 0.5, respectively. $M=1$ signifies the mean-field results. The many-body dynamics are computed with $M= 6$ time-dependent orbitals for $\Psi _G$ and $M= 10$ time-dependent orbitals for $\Psi _Y$. We show here dimensionless quantities. Color codes are explained in each panel.

Next, we consider the momentum variance per particle along the *x*-direction, $\dfrac{1}{N}\Delta _{{\hat{P}_X}}^2(t)$, and present its time evolution at the mean-field and many-body levels for $\Psi _G$ and $\Psi _Y$ in Fig. 6. We explicitly show the possible quantum correlations effects on the many-body dynamics of $\dfrac{1}{N}\Delta _{{\hat{P}_X}}^2(t)$ at the resonant tunneling condition. Recall that $\dfrac{1}{N}\Delta _{{\hat{P}_X}}^2(t)$ is relatively a more complex quantity than $\dfrac{1}{N}\Delta _{\hat{X}}^2(t)$ in the junction as the former one is controlled by and more sensitive to the shape of the orbitals. In Fig. 6, we observe, also for $\dfrac{1}{N}\Delta _{{\hat{P}_X}}^2(t)$, two types of oscillations, smaller amplitude with higher frequency and larger amplitude with lower frequency. But there is a difference with respect to $\dfrac{1}{N}\Delta _{\hat{X}}^2(t)$. For the symmetric double-well potential, $\dfrac{1}{N}\Delta _{{\hat{P}_X}}^2(t)$ is dominated by the breathing mode oscillations which produce small amplitude with high frequency oscillations (Fig. S1 of the supplemental materials). While at the first resonant tunneling, $c=0.25$, the density oscillations are more prominent producing the larger amplitude but lower frequency oscillations. But, at the second resonant tunneling, $c=0.5$, $\dfrac{1}{N}\Delta _{{\hat{P}_X}}^2(t)$ shows a mixture of the density and breathing mode oscillations. At $c=0.25$, the mean-field $\dfrac{1}{N}\Delta _{{\hat{P}_X}}^2(t)$ oscillates between 0.5 and 1.5, while at $c=0.5$, between 0.5 and 2.5. This reflects the ground (left well) and excited (right well) orbitals participating in the dynamics. One of the interesting features at the resonant tunneling condition is that the minima values of the many-body $\dfrac{1}{N}\Delta _{{\hat{P}_X}}^2(t)$ increases with the growing degree of fragmentation. Also, the amplitude of the many-body oscillations of $\dfrac{1}{N}\Delta _{{\hat{P}_X}}^2(t)$ eventually decays, which is clearly visible at $c=0.25$, when the density collapses. It suggests that in the long-time dynamics of $\dfrac{1}{N}\Delta _{{\hat{P}_X}}^2(t)$, the excited state in the right well slowly wins over the initial state prepared in the left well.

In this context, it is worthwhile to briefly discuss the position and momentum variances along the *y*-direction, $\dfrac{1}{N}\Delta _{{\hat{Y}}}^2(t)$ and $\dfrac{1}{N}\Delta _{{\hat{P}_Y}}^2(t)$, respectively. We found in our work that the mean-field and many-body values of $\dfrac{1}{N}\Delta _{{\hat{Y}}}^2(t)$ and $\dfrac{1}{N}\Delta _{{\hat{P}_Y}}^2(t)$ have very small fluctuations, of the order of $10^{-3}$, atop the value of the non-interacting bosons, i.e., 0.5 and 1.5 for both variances, for $\Psi _G$ and $\Psi _Y$, respectively (see supplemental materials). Also, with the change in asymmetry parameter, we do not observe any substantial changes in the values of position and momentum variances along the transverse direction. However, in addition to the motion along the *x*-direction, the effect of the seemingly constant transverse degrees-of-freedom lead to the existence of a purely two-dimensional quantity, such as the angular-momentum.

**Figure 7:**
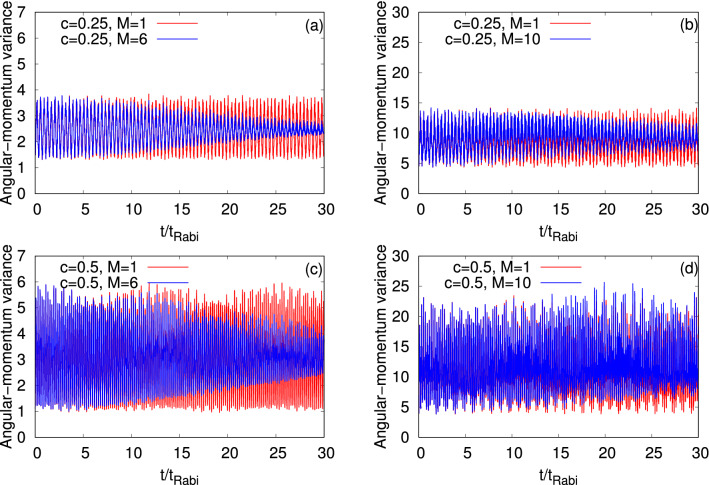
Time-dependent variance per particle of the *z*- component of the angular-momentum operator, $\dfrac{1}{N}\Delta _{\hat{L}_Z}^2(t)$, in a longitudinally-asymmetric 2D double-well potential for $\Psi _G$ (left column) and $\Psi _Y$ (right column) of $N=10$ bosons with the interaction parameter $\Lambda =0.01\pi$. The upper and lower rows show $\dfrac{1}{N}\Delta _{{\hat{L}_Z}}^2(t)$ at the resonant tunneling with asymmetry parameter, $c=0.25$ and 0.5, respectively. $M=1$ signifies the mean-field results. The many-body dynamics are computed with $M= 6$ time-dependent orbitals for $\Psi _G$ and $M= 10$ time-dependent orbitals for $\Psi _Y$. We show here dimensionless quantities. Color codes are explained in each panel.

Now, we examine the angular-momentum variance per particle, $\dfrac{1}{N}\Delta _{{\hat{L}_Z}}^2(t)$, and demonstrate the implications of the resonant tunneling on its dynamical behavior in Fig. 7, see Appendix A for the mathematical form of $\dfrac{1}{N}\Delta _{{\hat{L}_Z}}^2(t)$. The results are compared at the mean-field and many-body levels for $\Psi _G$ and $\Psi _Y$. At $t=0$, in case of the symmetric double-well trap, $\dfrac{1}{N}\Delta _{{\hat{L}_Z}}^2(t)$ can be calculated analytically and is found to be 2 and 7 for $\Psi _G$ and $\Psi _Y$, respectively. At the first and second resonant tunneling, the initial values of $\dfrac{1}{N}\Delta _{{\hat{L}_Z}}^2(t)$ for $\Psi _G$ are reduced to 1.5 and 1, respectively. But for $\Psi _Y$, $\dfrac{1}{N}\Delta _{{\hat{L}_Z}}^2(t)$ remains 7 at $t=0$ for the resonant values of *c*. The mean-field and many-body dynamics of $\dfrac{1}{N}\Delta _{{\hat{L}_Z}}^2(t)$ in the symmetric double-well potential for the states $\Psi _G$ and $\Psi _Y$ are demonstrated in Fig. S1 of the supplemental materials. For the symmetric double-well potential, we find that $\dfrac{1}{N}\Delta _{{\hat{L}_Z}}^2(t)$ of $\Psi _G$ oscillates with amplitude of fluctuations in the order of $10^{-1}$ at the mean-field level. A rather small difference, marking the collapse of the density oscillations, is present between the time evolutions of $\dfrac{1}{N}\Delta _{{\hat{L}_Z}}^2(t)$ at the mean-field and many-body levels. Unlike for $\Psi _G$, interesting many-body features are found for $\Psi _Y$ at $c=0$. It is noticed that the amplitude of the many-body $\dfrac{1}{N}\Delta _{{\hat{L}_Z}}^2(t)$ of $\Psi _Y$ initially grows and, as time passes by, it decays (at around $t=12t_{Rabi}$) for the symmetric double-well potential. The maximal fluctuations on top of the baseline of $\dfrac{1}{N}\Delta _{{\hat{L}_Z}}^2(t)$ at the many-body level is 34% where as at the mean-field level it is 7%. Contrary to the symmetric double-well potential, at resonant tunneling, the angular-momentum variance shows a sudden increase in the amplitude both for $\Psi _G$ and $\Psi _Y$. The mean-field and many-body dynamics of $\dfrac{1}{N}\Delta _{{\hat{L}_Z}}^2(t)$ are oscillatory in nature at the resonant tunneling values of *c*. At $c=0.25$, the mean-field $\dfrac{1}{N}\Delta _{{\hat{L}_Z}}^2(t)$ oscillates between 1.5 and about 3.5 for $\Psi _G$ and, between 5 and about 13 for $\Psi _Y$. At the second resonant tunneling, the amplitude of the mean-field of $\dfrac{1}{N}\Delta _{{\hat{L}_Z}}^2(t)$ further increases and oscillates between 1 and approximately 6 for $\Psi _G$, and between 5 and approximately 22 for $\Psi _Y$. We notice that $\Psi _Y$ has always larger fluctuations of amplitude of $\dfrac{1}{N}\Delta _{{\hat{L}_Z}}^2(t)$ compared to $\Psi _G$ at a fixed value of *c*. The many-body $\dfrac{1}{N}\Delta _{{\hat{L}_Z}}^2(t)$ overlaps with the corresponding mean-field results at short time scales and shows a decay in amplitude as fragmentation grows, which is more evident at $c=0.25$. The rate of decay of $\dfrac{1}{N}\Delta _{{\hat{L}_Z}}^2(t)$ values is slower for $\Psi _Y$, which is consistent with the survival probability and $\dfrac{1}{N}\Delta _{{\hat{P}_X}}^2(t)$.

Until now, we determined the effect of the transverse excitations on the dynamics of $\Psi _G$ and $\Psi _Y$ at the resonant tunneling condition within the mean-field and many-body levels of theory. The effects of the transverse degrees-of-freedom are analyzed in terms of various physical quantities. The results prove that the transverse direction have substantial influences on the dynamics of the ground as well as excited states at the longitudinal resonant tunneling scenario in two spatial dimensions.

### Transversal resonant tunneling

Proceeding to the transversal resonant tunneling, we want to emphasize that this scenario does only exist in 2D, thereby bringing to the front new degrees-of-freedom with the transverse direction, which we would like to explore. We have found that transverse excitations play a substantial role in longitudinal resonant tunneling. Here, we ask how and in what capacity longitudinal excitations play a role in transversal resonant tunneling.

In order to investigate the transversal resonant tunneling phenomenon, we assume the bosons are prepared either as the non-interacting, $\Psi _G=\dfrac{1}{\sqrt{\pi }}F(x,y)$, or longitudinally-excited state, $\Psi _X=\sqrt{\dfrac{2}{\pi }}(x+2)F(x,y)$, in the left well, $V_L^\prime (x,y)$, of a transversely-asymmetric 2D double-well potential, $V_T^\prime (x,y)$. Here the asymmetry means the right well is wider than the left well. This is generated by making the transverse frequency of the trap spatially-dependent. $V_L^\prime (x,y)$ is functioned as

5 $$\begin{aligned} V_L^\prime (x,y)= {\left\{ \begin{array}{ll} \dfrac{1}{2}(x+2)^2+\dfrac{1}{2}y^2,  x<-1, -\infty< y<\infty , \\ \dfrac{1}{2}(x+2)^2+\dfrac{1}{2}[S(x)]^2y^2,  -1< x<+1, -\infty< y<\infty , \\ \dfrac{1}{2}(x+2)^2+\dfrac{1}{2}\omega _n^2y^2,  x>+1, -\infty< y<\infty . \end{array}\right. } \end{aligned}$$

**Figure 8:**
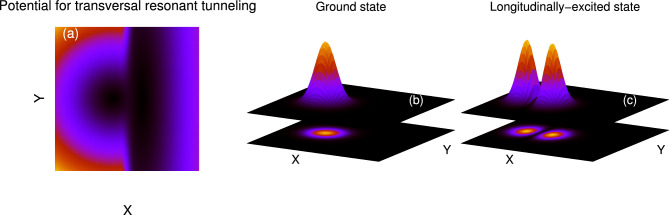
(**a**) Schematic diagrams for the asymmetric 2D double-well potential for transversal resonant tunneling, described in Eq. (6). Here the right well is wider than the left well. Panels (**b**) and (**c**) show the initial density distributions for the ground and longitudinally-excited states, respectively.

Here, $S(x)=\left[ 1+(\omega _n-1)sin^2\dfrac{(x+1)\pi }{4}\right]$ is a switching function for the transversal frequency, where $\omega _n$ is the minimal value of the transversal frequency. The initial density distributions of $\Psi _G$ and $\Psi _X$ are presented in Fig. 8. For investigating the time evolution, as described in the longitudinal resonant tunneling, we quench the inter-particle interaction at $t=0$ from $\Lambda =0$ to $\Lambda =0.01\pi$. At the same time, we convert $V_L^\prime (x,y)$ to the transversely-asymmetric 2D double-well potential $V_T^\prime (x,y)$ (see Fig. 8a), where

6 $$\begin{aligned} V_T^\prime (x,y)= {\left\{ \begin{array}{ll} \dfrac{1}{2}(x+2)^2+\dfrac{1}{2}y^2,  x<-1, -\infty< y<\infty , \\ \dfrac{1}{2}(x+2)^2+\dfrac{1}{2}[S(x)]^2y^2,  -1< x<-\dfrac{1}{2}, -\infty< y<\infty , \\ \dfrac{3}{2}(1-x^2)+\dfrac{1}{2}[S(x)]^2y^2,  |x|\le \dfrac{1}{2}, -\infty< y<\infty , \\ \dfrac{1}{2}(x-2)^2+\dfrac{1}{2}[S(x)]^2y^2,  \dfrac{1}{2}< x<1, -\infty< y<\infty , \\ \dfrac{1}{2}(x-2)^2+\dfrac{1}{2}\omega _n^2y^2,  x>+1, -\infty< y<\infty \end{array}\right. } \end{aligned}$$

Here the many-body computations are performed with $M=6$ time-dependent orbitals for both $\Psi _G$ and $\Psi _X$. For the numerical solution, the considered box size is $[-10, 10)\times [-10, 10)$ with periodic boundary conditions having the grid density $128\times 128$. Convergence of the results with respect to the number of orbitals and number of grid points have been checked and presented in the supplemental materials. The number of bosons and interaction parameter are chosen as $N=10$ and $\Lambda =0.01\pi$, same as in the case of longitudinal resonant tunneling. The mean-field dynamics are also computed with the interaction parameter $\Lambda$.

**Figure 9:**
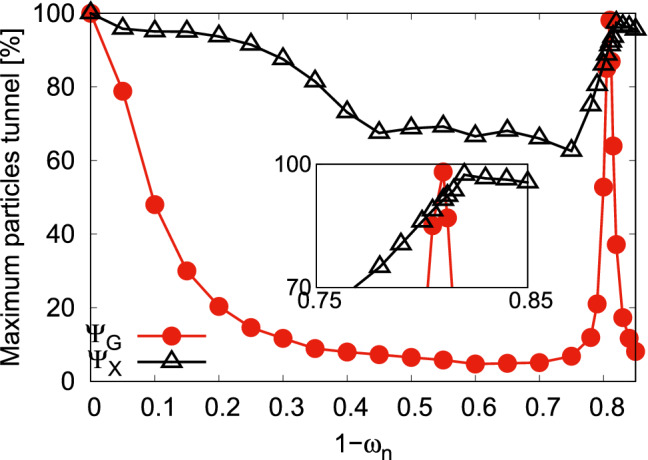
Variation of the maximal number of particles tunneling [in %] from the left to the right well with the trapping frequency $\omega _n$ of the right well for $\Psi _G$ and $\Psi _X$ (see Fig. 1b). The interaction parameter is $\Lambda =0.01\pi$. The same plot is highlighted in the inset by enlarging the resonance region. The data is generated from the time-dependent solution of the Gross-Pitaeveskii equation. We show here dimensionless quantities.

At first, we examine how the transversal resonant tunneling can be achieved for two initial bosonic structures, $\Psi _G$ and $\Psi _X$, in a transversely-asymmetric 2D double-well potential presented in Eq. (6). Here, we gradually decrease the transverse frequency in the right well, $\omega _n$, from an initial value ($\omega _n=1$) which represents a symmetric double-well potential. Similarly to the longitudinal resonant tunneling, here also, the initial states are prepared in the left well of the double-well potential. Figure 9 presents the maximal number of particles tunneling to the right well for $\Psi _G$ and $\Psi _X$. The maximal number is extracted from the time-dependent solution of the Gross-Pitaeveskii equation using $\int \limits _{x=0}^{+\infty }\int \limits _{y=-\infty }^{+\infty }dx dy \dfrac{\rho (x,y;t)}{N}$, where $\rho (x,y;t)$ is the density of the bosonic cloud. One can see from Fig. 9, at $\omega _n=1$, that the maximal number of particles tunneling to the right well is 100%. Decreasing the value of $\omega _n$ reduces the number of maximum particles which can tunnel to the right well for both initial bosonic clouds. The rate of decay of the maximal number of particles tunneling to the right well with $\omega _n$ is smaller for $\Psi _X$ as it essentially lies in the first excited band along the direction of the barrier and thus feels a smaller barrier when it tunnels. Further decrease of $\omega _n$, the maximal number of particles tunneling to the right well grows. When the one-body energy of the left well coincides with one of the one-body higher energy levels of the right well, again the maximal number of particles tunneling to the right well reaches to almost 100%. We find only one resonant tunneling for the span of $\omega _n$ considered here. For $\Psi _G$ and $\Psi _X$, the transversal resonant tunneling occurs at $\omega _n=0.19$ and $\omega _n=0.18$, respectively. Also, it can be observed from the figure that for slightly off-resonant values of $\omega _n$, the maximal number of particles tunneling to the right well significantly decreases in comparison to the respective value of $\omega _n$ at resonant tunneling for $\Psi _G$, but it varies much weaker with $\omega _n$ for $\Psi _X$.

**Figure 10:**
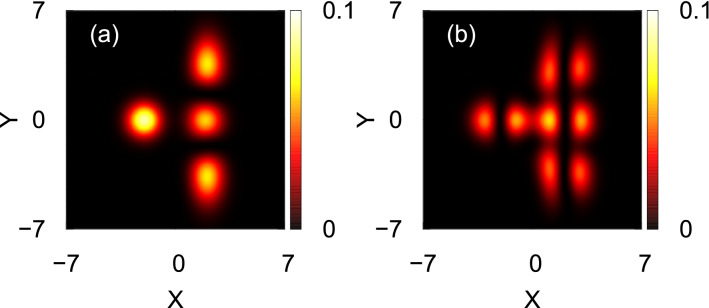
The densities per particle for the initial states (**a**) $\Psi _G$ and (**b**) $\Psi _X$. The interaction parameter is $\Lambda =0.01\pi$. The plots are generated at $t\approx t_{Rabi}$ from the time-dependent solution of the Gross-Pitaeveskii equation. We show here dimensionless quantities.

The transversal resonant tunneling for $\Psi _G$ found in Fig. 9 is when $\Psi _G$ of the left well couples to $(y^2-1)\Psi _G$ of the right well. Similarly, $\Psi _X$ of the left well couples to $(y^2-1)\Psi _X$ of the right well to facilitate the transversal resonant tunneling of $\Psi _X$, see Fig. 10. The resonant tunneling channels $\Psi _G \rightarrow y\Psi _G$ and $\Psi _X \rightarrow y\Psi _X$ are symmetry forbidden, at least at the mean-field level, as the time-dependent orbital becomes odd with respect to $y\rightarrow -y$. Here we select three values of $\omega _n$ which are either at the resonant tunneling value or close to it, say, $\omega _n=0.18$, 0.19, and 0.20, and investigate the overall dynamical response through the survival probability, loss of coherence, and the variances of position, momentum, and angular-momentum operators. To show the effects of the many-body correlations on different physical quantities, we compare the results of many-body dynamics with corresponding results at the mean-field level.

**Figure 11:**
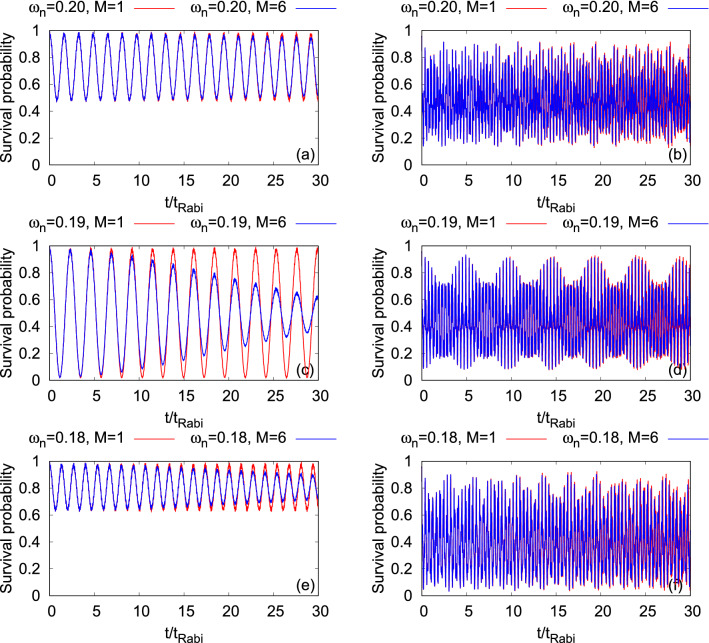
Dynamics of the survival probability of $\Psi _G$ (left column) and $\Psi _X$ (right column) in the left well of a transversely-asymmetric 2D double-well potential of $N=10$ bosons with the interaction parameter $\Lambda =0.01\pi$. First, second, and third rows show the results at frequency, $\omega _n=0.20$, 0.19, and 0.18, respectively. $M=1$ signifies the mean-field results. The many-body dynamics are computed with $M= 6$ time-dependent orbitals. We show here dimensionless quantities. Color codes are explained in each panel.

Now, we start with the discussion of the density oscillations in terms of the survival probability in the left well for both the states $\Psi _G$ and $\Psi _X$. Figure 11c and f shows the dynamical behaviors of the survival probabilities at the resonant tunneling conditions for $\Psi _G$ and $\Psi _X$, respectively. We notice that the frequencies of tunneling of the density between the left and right wells are different for $\Psi _G$ but are essentially same for $\Psi _X$ for different $\omega _n$ considered here. At $\omega _n=0.18$, 0.19, and 0.20, the tunneling frequencies for $\Psi _G$ are 1.41$t_{Rabi}$, 2.30$t_{Rabi}$, and 1.55$t_{Rabi}$, respectively. While for $\Psi _X$, the tunneling frequency is around 0.38$t_{Rabi}$ for all three $\omega _n$ values.

At a fixed value of $\omega _n$, both the frequency and amplitude of the survival probability at the mean-field and many-body levels are practically the same for $\Psi _X$. Whereas for $\Psi _G$, although the frequency of the survival probability are very similar at the mean-field and many-body levels, the amplitudes of the survival probability show a significant deviation which is a maximal at $\omega _n=0.19$. The gradual decay at the many-body level of dynamics of the survival probability signifies the growing degree of quantum correlations which will be quantitatively discussed later in terms of the loss of coherence in the system. All in all, Fig. 11 reflects that correlations develops faster for $\Psi _G$ compared to $\Psi _X$.

**Figure 12:**
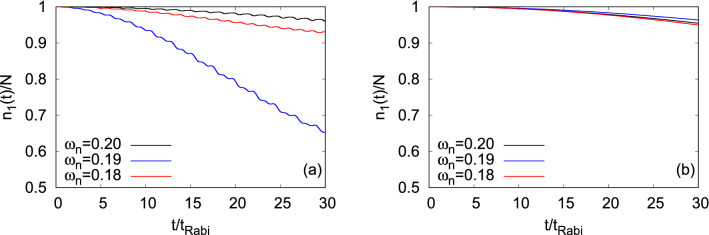
Time-dependent occupation per particle of the first natural orbital, $\dfrac{n_1(t)}{N}$, in a transversely-asymmetric 2D double-well potential. The number of bosons is $N=10$ and the interaction parameter $\Lambda =0.01\pi$. Panels (**a**) and (**b**) are for $\Psi _G$ and $\Psi _X$, respectively. The data have been obtained with $M = 6$ time-dependent orbitals. We show here dimensionle ss quantities. Color codes are explained in each panel.

Now, we demonstrate the effect of growing degree of correlations on the loss of coherence of the bosonic clouds, $\Psi _G$ and $\Psi _X$, at the transversal resonant tunneling and also at its vicinity. In Fig. 12, we display the time-dependent occupation of the first natural orbital, namely the condensate fraction, $\dfrac{n_1(t)}{N}$. As observed in the longitudinal resonant tunneling, here also, $\dfrac{n_1(t)}{N}$ decays with time with a weak oscillatory background. As $\Psi _G$ lies in the lowest band, it feels the barrier more and yields comparatively strong background oscillations than $\Psi _X$. It is observed that both states lose their coherence faster at the resonant value of $\omega _n$ and the loss of coherence becomes gradually slower away from the resonant tunneling. The loss of coherence at the resonant tunneling are seen to be almost 35% and 5% for $\Psi _G$ and $\Psi _X$, respectively, at the maximum time ($t=30t_{Rabi}$) considered here. Side by side, the loss of coherence in the system is accompanied by a growing degree of fragmentation, leading to increase of the occupancy of higher natural orbitals. See the supplemental materials for the detailed analysis of the mechanism of fragmentation in transversal resonant tunneling as well as to verification of convergence.

To further characterize the many-body correlations at the transversal resonant tunneling condition for $\Psi _G$ and $\Psi _X$, we graphically present the dynamical behavior of the variances of the position and momentum operators along the *x*- and *y*-directions, and the angular-momentum operator. Also, the many-body results are compared with the corresponding mean-field results. These physical quantities at other values of $\omega _n$, i.e., 0.18 and 0.20 for $\Psi _G$ and 0.19 and 0.20 for $\Psi _X$, along with their convergences are demonstrated in the supplemental materials.

**Figure 13:**
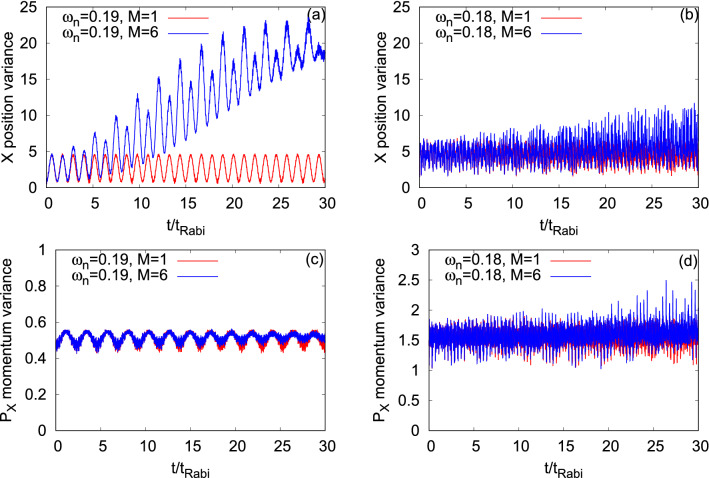
Time-dependent position and momentum variances per particle along the *x*-direction in a transversely-asymmetric 2D double-well potential for $\Psi _G$ (left column, $\omega _n=0.19$) and $\Psi _X$ (right column, $\omega _n=0.18$) under transversal resonant tunneling conditions. The number of bosons is $N=10$ and the interaction parameter $\Lambda =0.01\pi$. $M=1$ signifies the mean-field results. The many-body dynamics are computed with $M= 6$ time-dependent orbitals. We show here dimensionless quantities. Color codes are explained in each panel.

Figure 13 displays the dynamics of the many-particle position and momentum variances per particle along the *x*-direction, $\dfrac{1}{N}\Delta _{\hat{X}}^2(t)$ and $\dfrac{1}{N}\Delta _{{\hat{P}_X}}^2(t)$, respectively, at the transversal resonant tunneling conditions of $\Psi _G$ and $\Psi _X$. Details of $\dfrac{1}{N}\Delta _{\hat{X}}^2(t)$ and $\dfrac{1}{N}\Delta _{{\hat{P}_X}}^2(t)$ for $\Psi _G$ and $\Psi _X$ in the symmetric double-well potential are discussed in Fig. S1 of the supplemental materials. The panels show that the fragmentation developed in the system yields the deviation of the many-body results compared to the corresponding mean-field one. This deviation is larger for $\Psi _G$ in comparison with $\Psi _X$, which is consistent with the corresponding survival probability. For $\Psi _G$, the many-body dynamics of $\dfrac{1}{N}\Delta _{\hat{X}}^2(t)$ and $\dfrac{1}{N}\Delta _{{\hat{P}_X}}^2(t)$ almost reach at its equilibrium when the density oscillations collapse. It is found that the frequency of oscillations is practically the same for the mean-field and many-body dynamics of $\dfrac{1}{N}\Delta _{\hat{X}}^2(t)$ and $\dfrac{1}{N}\Delta _{{\hat{P}_X}}^2(t)$. As we have seen in the dynamics of the survival probability, the oscillation frequency of both quantities is higher for $\Psi _X$ compared to $\Psi _G$. Moreover, both for $\Psi _G$ and $\Psi _X$, we find that the frequency of oscillations of the survival probability is twice the frequency of oscillations of $\dfrac{1}{N}\Delta _{\hat{X}}^2(t)$ while they are practically identical for $\dfrac{1}{N}\Delta _{{\hat{P}_X}}^2(t)$. An interesting many-body feature is observed for $\Psi _G$ in the time evolution of $\dfrac{1}{N}\Delta _{\hat{X}}^2(t)$. Here, we find that there are two types of oscillations taking place, namely, one with high amplitude and the second with a small amplitude. This type of oscillations may arise due to the transition of bosons from the lower to higher band, which requires a detailed analysis. Many-body linear-response theory^94^, is capable of identifying many-body excitation spectrum of interacting bosons, which goes beyond the scope of the present work. The time evolution of $\dfrac{1}{N}\Delta _{{\hat{P}_X}}^2(t)$ is accompanied by two types of oscillations, smaller amplitude with higher frequency and larger amplitude with lower frequency, but is mostly dominated by the former one which is coming from the breathing mode oscillations.

**Figure 14:**
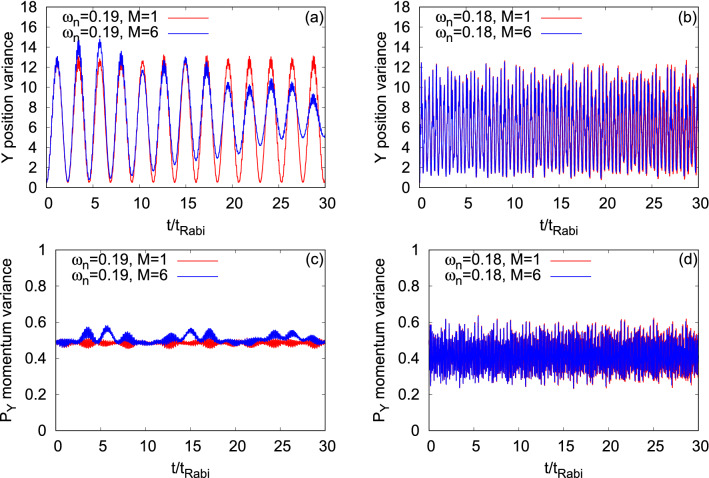
Time-dependent position and momentum variance per particle along the *y*-direction in a transversely-asymmetric 2D double-well potential for $\Psi _G$ (left column, $\omega _n=0.19$) and $\Psi _X$ (right column, $\omega _n=0.18$) under transversal resonant tunneling conditions. The number of bosons is $N=10$ and the interaction parameter $\Lambda =0.01\pi$. $M=1$ signifies the mean-field results. The many-body dynamics are computed with $M= 6$ time-dependent orbitals. We show here dimensionless quantities. Color codes are explained in each panel.

Now, let us discuss the role played by the transversal resonant tunneling of $\Psi _G$ and $\Psi _X$ on the time evolution of the position and momentum variances along the *y*-direction, $\dfrac{1}{N}\Delta _{{\hat{Y}}}^2(t)$ and $\dfrac{1}{N}\Delta _{{\hat{P}_Y}}^2(t)$, respectively (see Fig. 14). Unlike in the longitudinal resonant tunneling and in the case of symmetric double-well potential where the position and momentum variances along the *y*-direction of $\Psi _G$ and $\Psi _X$ have very small fluctuations in amplitude (of the order of 10^−3^, see Fig. S4 and discussion of the first section in the supplemental materials), here, in the transversal resonant tunneling, we observe comparatively large fluctuations which are clearly more prominent for $\dfrac{1}{N}\Delta _{{\hat{Y}}}^2(t)$. For both the states, $\Psi _G$ and $\Psi _X$, it is found that the frequency of oscillation of $\dfrac{1}{N}\Delta _{{\hat{Y}}}^2(t)$ overlaps with the corresponding frequency of the survival probability. The amplitude of the many-body time evolution of $\dfrac{1}{N}\Delta _{{\hat{Y}}}^2(t)$ decays with time, originating from the growing degree of fragmentation which is more evident for $\Psi _G$. It is clear from Fig. 14 that $\dfrac{1}{N}\Delta _{{\hat{Y}}}^2(t)$ is dominated by the density oscillations while $\dfrac{1}{N}\Delta _{{\hat{P}_Y}}^2(t)$ by the breathing oscillations. We notice that, for $\Psi _G$, the transversal resonant tunneling gives rise to a beating pattern in the many-body dynamics of $\dfrac{1}{N}\Delta _{{\hat{Y}}}^2(t)$ and $\dfrac{1}{N}\Delta _{{\hat{P}_Y}}^2(t)$. This beating pattern may be the consequence of the combination of different breathing frequencies. A dedicated study of many-body excitations in the transversely-asymmetric 2D double-well potential could resolve the many-boson states.

**Figure 15:**
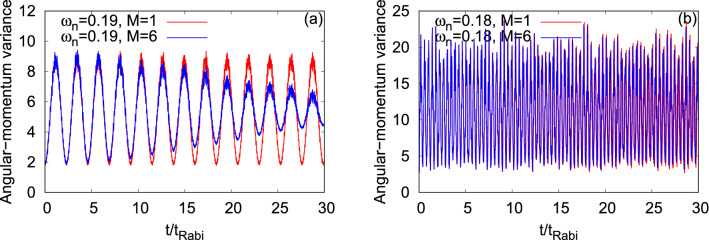
Time-dependent variance per particle of the *z*- component of the angular-momentum operator, $\dfrac{1}{N}\Delta _{\hat{L}_Z}^2(t)$, in a transversely-asymmetric 2D double-well potential for $\Psi _G$ (left column, $\omega _n=0.19$) and $\Psi _X$ (right column, $\omega _n=0.18$) under transversal resonant tunneling conditions. The number of bosons is $N=10$ and the interaction parameter $\Lambda =0.01\pi$. $M=1$ signifies the mean-field results. The many-body dynamics are computed with $M= 6$ time-dependent orbitals. We show here dimensionless quantities. Color codes are explained in each panel.

Proceeding, we examine the time evolution of the angular-momentum variance per particle, $\dfrac{1}{N}\Delta _{{\hat{L}_Z}}^2(t)$, for $\Psi _G$ and $\Psi _X$ at the transversal resonant tunneling scenario (see Fig. 15). For the dynamics of $\dfrac{1}{N}\Delta _{{\hat{L}_Z}}^2(t)$ in a symmetric double-well potential for $\Psi _G$ and $\Psi _X$, we refer to the discussion of Fig. S4 of the supplemental materials. The mean-field dynamics of $\dfrac{1}{N}\Delta _{{\hat{L}_Z}}^2(t)$ for $\Psi _G$ showcases smooth undulations and it is uneven for $\Psi _X$ which is consistent with the survival probabilities of the corresponding states. The amplitude of the oscillations of the mean-field $\dfrac{1}{N}\Delta _{{\hat{L}_Z}}^2(t)$ of $\Psi _X$ is larger compared to $\Psi _G$, as one can see in panels (a) and (b) of Fig. 15. For both states, we notice that the frequency of the oscillations of $\dfrac{1}{N}\Delta _{{\hat{L}_Z}}^2(t)$ overlaps with the corresponding frequency of $\dfrac{1}{N}\Delta _{{\hat{Y}}}^2(t)$. The growing degree of the fragmentation causes a decay in amplitude of the many-body $\dfrac{1}{N}\Delta _{{\hat{L}_Z}}^2(t)$ which is significantly more prominent for $\Psi _G$ and hardly visible for $\Psi _X$.

## Summary and conclusions

The paradigm of a bosonic Josephson junction, in which bosons can tunnel back and forth between two potential wells, is shown to be very rich at resonant tunneling condition in two spatial dimensions. We have investigated the dynamical behavior of a few intricate coherent bosonic clouds at resonant tunneling conditions in a two-dimensional asymmetric Josephson junction. In particular, we examine two types of resonant tunnelings, longitudinal and transversal. For the longitudinal resonant tunneling, the initial bosonic clouds are considered as the ground and transversely-excited states, whereas for transversal resonant tunneling, they are taken as the ground and longitudinally-excited states. The excited states are considered in such a way that we can explore the impact of longitudinal resonant tunneling on the transversely-excited state and analogously, transversal resonant tunneling on the longitudinally-excited state. The dynamical behavior is analyzed by solving the full many-body Schrödinger equation and presenting the time evolution of the survival probability, depletion or fragmentation, and the many-particle position, momentum, and angular momentum variances. To characterize the influence of the many-body correlations on the tunneling dynamics of the different shapes of the bosonic clouds, we compare the mean-field and many-body results.

Focusing on the longitudinal resonant tunneling scenario, we verify that a gradual change of asymmetry parameter *c* either from the values of resonant tunnelings, or the value for which one gets the symmetric double-well potential, decreases the tunneling probability of the bosons. The many-body correlations exhibit the collapse of the density oscillations which is manifested in the dynamics of the survival probability. The collapse rate becomes slower when one moves away from the symmetric double-well potential to the first longitudinal resonant tunneling condition ($c=0.25$) and further slower toward the second longitudinal resonant tunneling condition ($c=0.5$). Moreover, at $c=0.5$ we find a partial revival pattern in the mean-field as well as the many-body survival probabilities. Although the rate of collapse of density oscillation for $\Psi _Y$ is slower compared to $\Psi _G$ at a fixed value of *c*, the presence of transverse excitation in $\Psi _Y$ makes it more fragmented than $\Psi _G$ at a given time. Compared to the symmetric double-well potential, we observe that both initial states become less fragmented at $c=0.25$ and further less fragmented at $c=0.5$.

Referring to the transversal resonant tunneling scenario, we scan the frequency along the transverse direction in the right well, $\omega _n$, starting from the value $\omega _n=1$ (symmetric 2D double-well) and reaching down to $\omega _n=0.17$, and find that the first resonant tunneling in the transverse direction occurs at $\omega _n=0.19$ and 0.18 for $\Psi _G$ and $\Psi _X$, respectively. This resonant tunneling is achieved when the bosons tunnel from $\Psi _G$ of the left well to $(y^2-1)\Psi _G$ of the right well. Similarly, for $\Psi _X$, the bosons tunnel from $\Psi _X$ to $(y^2-1)\Psi _X$. The time evolution of the many-body survival probability of $\Psi _G$ and $\Psi _X$ signifies that the rate of collapse of density oscillations is faster for $\Psi _G$ compared to $\Psi _X$. This collapse of density oscillations occurs due to the growing degree of many-body correlations which is graphically shown in terms of the loss of coherence in the system.

To obtain further information of the time-dependent many-particle wavefunction, we present the many-particle variances of the observables, $\dfrac{1}{N}\Delta _{{\hat{X}}}^2(t)$, $\dfrac{1}{N}\Delta _{{\hat{Y}}}^2(t)$, $\dfrac{1}{N}\Delta _{{\hat{P}_X}}^2(t)$, $\dfrac{1}{N}\Delta _{{\hat{P}_Y}}^2(t)$, and $\dfrac{1}{N}\Delta _{{\hat{L}_Z}}^2(t)$, both at the longitudinal and transversal resonant tunneling conditions. Also, we demonstrate a possible interconnection of the above-mentioned quantities with the survival probability and loss of coherence. It is observed that the growing degree of fragmentation impacts the time-evolution of each variance for the longitudinal and transversal resonant tunneling differently. We notice that, in general, at the resonant tunneling, effects of breathing dynamics are amplified in the dynamics of variances. Focusing on the longitudinal resonant tunneling at $c=0.5$, a signature of partial revival is observed in the dynamics of $\dfrac{1}{N}\Delta _{{\hat{X}}}^2(t)$, $\dfrac{1}{N}\Delta _{{\hat{P}_X}}^2(t)$, and $\dfrac{1}{N}\Delta _{{\hat{L}_Z}}^2(t)$. Also, in the longitudinal resonant tunneling, $\dfrac{1}{N}\Delta _{{\hat{Y}}}^2(t)$ shows a very small-amplitude fluctuations of the order of $10^{-3}$, which is contrary to the transversal resonant tunneling where the amplitude of $\dfrac{1}{N}\Delta _{{\hat{Y}}}^2(t)$ is significantly high, of the order of $10^1$. Moreover, the breathing oscillations in the dynamics of $\dfrac{1}{N}\Delta _{{\hat{P}_Y}}^2(t)$ at the longitudinal resonant tunneling is barely visible, while at the transversal resonant tunneling it is appreciable. Interestingly, at the transversal resonant tunneling, we notice a beating pattern in the dynamics of $\dfrac{1}{N}\Delta _{{\hat{Y}}}^2(t)$ and $\dfrac{1}{N}\Delta _{{\hat{P}_Y}}^2(t)$ which may arise due to the mixture of different breathing frequencies.

The present work can inspire several promising and interesting future research directions. An immediate extension would be the dynamics of even more intricate bosonic structures, e.g., vortex state or a mixture of vortex states induced by Raman process using light with orbital angular momentum^87–92^ or by synthetic magnetic fields^93^ in the longitudinal and transversal resonant tunneling conditions. Also, to apply a linear response theory^94^ to accurately calculate the different breathing frequencies involved in the time evolution of different quantities at the resonant tunneling scenario would be challenging. Finally, in the long run we could envision that additional geometries would open up in three spatial dimensions.

## Supplementary Information

Supplementary Information 1.

Supplementary Information 2.
