# Supplementary material for: Longitudinal and transversal resonant tunneling of interacting bosons in a two-dimensional Josephson junction
Source: Sci Rep. 2022 Jan 12;12:627. doi: 10.1038/s41598-021-04312-6 (PMC8755734; doi:10.1038/s41598-021-04312-6)
Supplement: Supplementary file 1 — Supplementary Information 1. [file 41598_2021_4312_MOESM1_ESM.pdf]

Appendices for Longitudinal and transversal resonant tunneling of interacting bosons in a two-dimensional Josephson junction

Anal Bhowmik[1, 2, *] and Ofir E. Alon[1, 2]

[1]*Department of Mathematics, University of Haifa, Haifa 3498838, Israel*

[2]*Haifa Research Center for Theoretical Physics and Astrophysics, University of Haifa, Haifa 3498838, Israel*

APPENDIX A: MANY-PARTICLE VARIANCE

The variance of an observable, $\hat{A}$, is determined by the combination of the expectation values of $\hat{A}$ and $\hat{A}^2$. Here the expectation value of $\hat{A} = \sum_{j=1}^{N} \hat{a}(r_j)$ depends on the one-body operators while the expectation of $\hat{A}^2$ is a mixture of one- and two-body operators, $\hat{A}^2 = \sum_{j=1}^{N} \hat{a}^2(r_j) + \sum_{j<k} 2\hat{a}(r_j)\hat{a}(r_k)$. The variance can be written as [1]

$$\begin{aligned}\frac{1}{N}\Delta^2_{\hat{A}}(t) &= \frac{1}{N}[\langle\Psi(t)|\hat{A}^2|\Psi(t)\rangle - \langle\Psi(t)|\hat{A}|\Psi(t)\rangle^2]\\ &= \frac{1}{N}\Bigg\{\sum_j n_j(t)\int d\mathbf{r}\phi^*_j(\mathbf{r};t)\hat{a}^2(\mathbf{r})\phi_j(\mathbf{r};t) - \left[\sum_j n_j(t)\int d\mathbf{r}\phi^*_j(\mathbf{r};t)\hat{a}(\mathbf{r})\phi_j(\mathbf{r};t)\right]^2\\ &+ \sum_{jpkq}\rho_{jpkq}(t)\left[\int d\mathbf{r}\phi^*_j(\mathbf{r};t)\hat{a}(\mathbf{r})\phi_k(\mathbf{r};t)\right]\left[\int d\mathbf{r}\phi^*_p(\mathbf{r};t)\hat{a}(\mathbf{r})\phi_q(\mathbf{r};t)\right]\Bigg\},\end{aligned} \quad \text{(A.1)}$$

where $\{\phi_j(\mathbf{r};t)\}$ are the natural orbitals, $\{n_j(t)\}$ the natural occupations, and $\rho_{jpkq}(t)$ are the elements of the reduced two-particle density matrix, $\rho(\mathbf{r}_1,\mathbf{r}_2,\mathbf{r}'_1,\mathbf{r}'_2;t) = \sum_{jpkq}\rho_{jpkq}(t)\phi^*_j(\mathbf{r}'_1;t)\phi^*_p(\mathbf{r}'_2;t)$ $\phi_k(\mathbf{r}_1;t)\phi_q(\mathbf{r}_2;t)$. For one-body operators which are local in position space, the variance described in Eq A.1 boils down to [2]

$$\frac{1}{N}\Delta^2_{\hat{A}}(t) = \int d\mathbf{r}\frac{\rho(\mathbf{r};t)}{N}\hat{a}^2(\mathbf{r}) - N\left[\int \frac{\rho(\mathbf{r};t)}{N}\hat{a}(\mathbf{r})\right]^2 + \int d\mathbf{r}_1 d\mathbf{r}_2\frac{\rho^{(2)}(\mathbf{r}_1,\mathbf{r}_2,\mathbf{r}_1,\mathbf{r}_2;t)}{N}a(\mathbf{r}_1)a(\mathbf{r}_2), \quad \text{(A.2)}$$

where $\rho(\mathbf{r};t)$ is the time-dependent density. Eq A.1 describes the variances when the center-of-mass of the bosonic clouds are at the origin and the wavefunction is $\Psi(0,0)$ (or denoted in short by Ψ). As the initial states considered in this work are prepared at the position $(a,b) = (-2,0)$, here we

* abhowmik@campus.haifa.ac.il

present a general form of variances by incorporating the translated wavefunction, i.e., $\Psi(a,b)$ (or denoted in short by Ψ_{ab}). For the position and momentum operators, the variances do not change due to the translated wavefunction [1], therefore, $\frac{1}{N}\Delta^2_{\hat{X}}\Big|_{\Psi_{ab}} = \frac{1}{N}\Delta^2_{\hat{X}}\Big|_{\Psi}$, $\frac{1}{N}\Delta^2_{\hat{Y}}\Big|_{\Psi_{ab}} = \frac{1}{N}\Delta^2_{\hat{Y}}\Big|_{\Psi}$, $\frac{1}{N}\Delta^2_{\hat{P}_X}\Big|_{\Psi_{ab}} = \frac{1}{N}\Delta^2_{\hat{P}_X}\Big|_{\Psi}$, and $\frac{1}{N}\Delta^2_{\hat{P}_Y}\Big|_{\Psi_{ab}} = \frac{1}{N}\Delta^2_{\hat{P}_Y}\Big|_{\Psi}$. But, for the angular-momentum variance, the situation becomes intricate and expressed as [1]

$$\begin{aligned}\frac{1}{N}\Delta^2_{\hat{L}_Z}\Big|_{\Psi_{ab}} &= \frac{1}{N}\Delta^2_{\hat{L}_Z}\Big|_{\Psi} + \frac{1}{N}a^2\Delta^2_{\hat{P}_Y}\Big|_{\Psi} + \frac{1}{N}b^2\Delta^2_{\hat{P}_X}\Big|_{\Psi} \\ &+ \frac{1}{N}\Big\{a[\langle\Psi|\hat{L}_Z\hat{P}_Y + \hat{P}_Y\hat{L}_Z|\Psi\rangle - 2\langle\Psi|\hat{L}_Z|\Psi\rangle\langle|\Psi|\hat{P}_Y|\Psi\rangle] \\ &- b[\langle\Psi|\hat{L}_Z\hat{P}_X + \hat{P}_X\hat{L}_Z|\Psi\rangle - 2\langle\Psi|\hat{L}_Z|\Psi\rangle\langle|\Psi|\hat{P}_X|\Psi\rangle] \\ &- 2ab[\langle\Psi|\hat{P}_Y\hat{P}_X|\Psi\rangle - \langle\Psi|\hat{P}_Y|\Psi\rangle\langle|\Psi|\hat{P}_X|\Psi\rangle]\Big\}. \end{aligned} \tag{A.3}$$

Eq. (A.3) is used in the main text to evaluate the angular-momentum variance of the different initial conditions. For a wavefunction Ψ with particular spatial symmetry the evaluation of (A.3) can simplify.

APPENDIX B: ROLE OF THE WIDTH OF INTERPARTICLE INTERACTION POTENTIAL ON THE DYNAMICS

We have presented an extensive investigation leading to be a wealth of results on the physics of longitudinal and transversal resonant tunneling of a many-particle system in two spatial dimensions, both at the mean-field and many-body levels of theory. We have used a model potential of finite width and find it instructive and important to demonstrate the robustness of our findings to this parameter. In order to verify whether the width of the interparticle interaction potential, σ, affects the dynamical behavior at the longitudinal and transversal resonant conditions, we recomputed all the properties discussed in this work for two additional smaller widths, $\sigma = 0.25/\sqrt{\pi}$ and $\sigma = 0.25$, both at the mean-field and many-body levels. It is noted that the main text explores the dynamics with $\sigma = 0.25\sqrt{\pi}$ at both the resonant tunneling scenarios. In the mean-field dynamics, we find that the dynamical behavior of all the properties obtained for $\sigma = 0.25/\sqrt{\pi}$ and $\sigma = 0.25$ fall on top of the corresponding results computed for $\sigma = 0.25\sqrt{\pi}$. As for an example, we plot the dynamics of a sensitive quantity, $\frac{1}{N}\Delta^2_{\hat{X}}(t)$, see Fig. B1. The results for both resonant scenarios manifest that all the mean-field quantities discussed in this work are independent of σ.

To present the role of width of σ at the many-body dynamics, we select the dynamical behavior of the most sensitive quantities of the ground state at the first longitudinal resonant condition

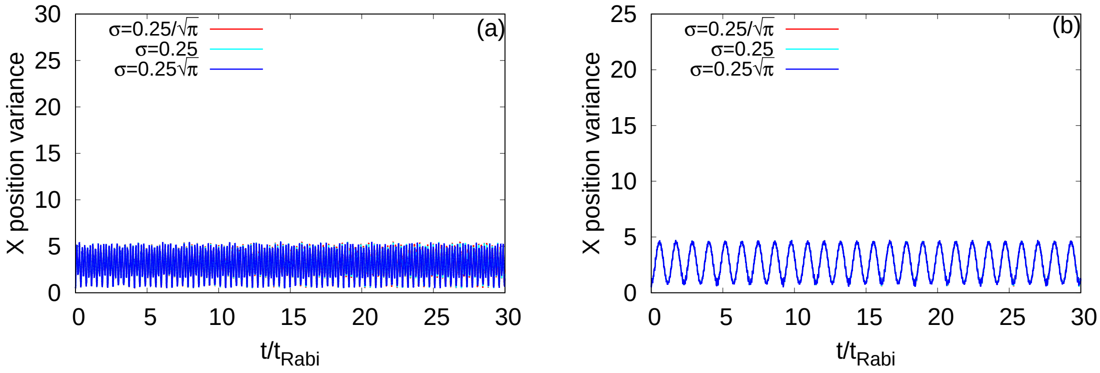

FIG. B1. Time-dependent mean-field position variance per particle along the x-direction, $\frac{1}{N}\Delta^2_{\hat{X}}(t)$, for the ground state at the (a) longitudinal ($c = 0.25$) and (b) transversal ($\omega_n = 0.19$) resonant scenarios. The three different widths of interparticle interaction potential are $\sigma = 0.25/\sqrt{\pi}$, $\sigma = 0.25$, and $\sigma = 0.25\sqrt{\pi}$. The interaction parameter is $\Lambda = 0.01\pi$. The mean-field results are found to be independent on σ. We show here dimensionless quantities. Color codes are explained in each panel.

($c = 0.25$) and transversal resonant condition ($\omega_n = 0.19$). In Fig. B2, we present the many-body dynamics of $\frac{n_1(t)}{N}$, $\frac{1}{N}\Delta^2_{\hat{X}}(t)$, $\frac{1}{N}\Delta^2_{\hat{P}_X}(t)$, and $\frac{1}{N}\Delta^2_{\hat{L}_Z}(t)$ at $c = 0.25$. From Fig. B2 (a), one can observe that the rate of loss of coherence is slightly faster for $\sigma = 0.25/\sqrt{\pi}$ compared to other values of σ. It is found that decreasing the width of σ of the interparticle interaction potential gradually diminishes the quantitative difference between the dynamical behaviors of a particular quantity. Interestingly, the variances which are known to be sensitive many-body quantities due to depletion are hardly influenced by the width of σ. Although there is a small quantitative difference in the long-time fragmentation dynamics for the choices of σ, the many-body physics described for the longitudinal resonant scenario is robust which can be seen in the dynamical behavior of the different quantum mechanical properties, see Fig. B2 (b), (c), and (d). Fig. B3 displays the role of σ at the transversal resonant scenario of the ground state. Here we present the dynamics of $\frac{n_1(t)}{N}$, $\frac{1}{N}\Delta^2_{\hat{X}}(t)$, $\frac{1}{N}\Delta^2_{\hat{Y}}(t)$ $\frac{1}{N}\Delta^2_{\hat{P}_X}(t)$, $\frac{1}{N}\Delta^2_{\hat{P}_Y}(t)$, and $\frac{1}{N}\Delta^2_{\hat{L}_Z}(t)$. Similar to the longitudinal resonant scenario, here also, we notice that the development of the fragmentation is slightly quicker for $\sigma = 0.25/\sqrt{\pi}$ and slightly slower for $\sigma = 0.25\sqrt{\pi}$. Moreover, there is no qualitative difference in the tunneling dynamics at the transversal resonant scenario for the different choices of σ. The beating pattern in the dynamics of $\frac{1}{N}\Delta^2_{\hat{P}_Y}(t)$ is also observed at $\sigma = 0.25/\sqrt{\pi}$ and $\sigma = 0.25$. All in all, we demonstrate and observe that the width of the interparticle interaction potential does not qualitatively affect the mean-field and many-body physics of the tunneling dynamics at the resonant tunneling scenario.

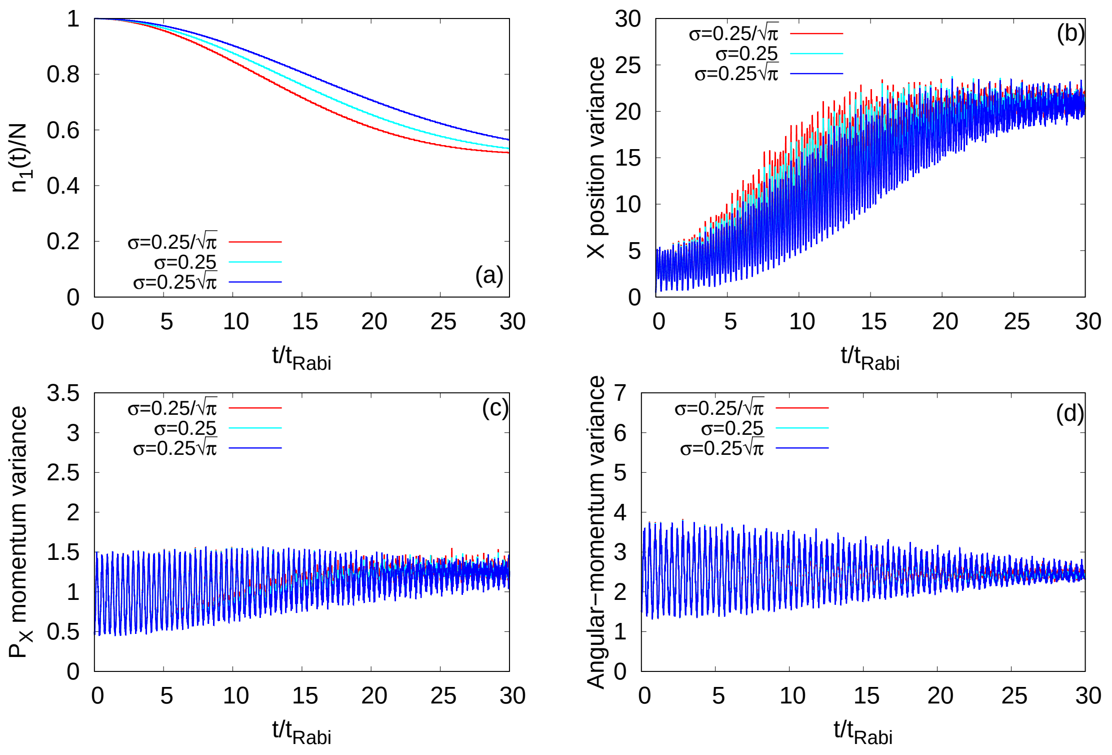

FIG. B2. Dependency of the many-body dynamics of ground state at the longitudinal resonant condition, $c = 0.25$, for the three different widths of interparticle interaction potential, i.e., $\sigma = 0.25/\sqrt{\pi}$, $\sigma = 0.25$, and $\sigma = 0.25\sqrt{\pi}$. The dynamics is shown by (a) $\frac{n_1(t)}{N}$, (b) $\frac{1}{N}\Delta^2_{\hat{X}}(t)$, (c) $\frac{1}{N}\Delta^2_{\hat{P}_X}(t)$, and (d) $\frac{1}{N}\Delta^2_{\hat{L}_Z}(t)$. The dynamics are computed with $N = 10$ bosons and the interaction parameter $\Lambda = 0.01\pi$. The many-body dynamics are computed with $M = 6$ time-dependent orbitals. The quantitative many-body results are found to be weakly dependent on σ. with We show here dimensionless quantities. Color codes are explained in each panel.

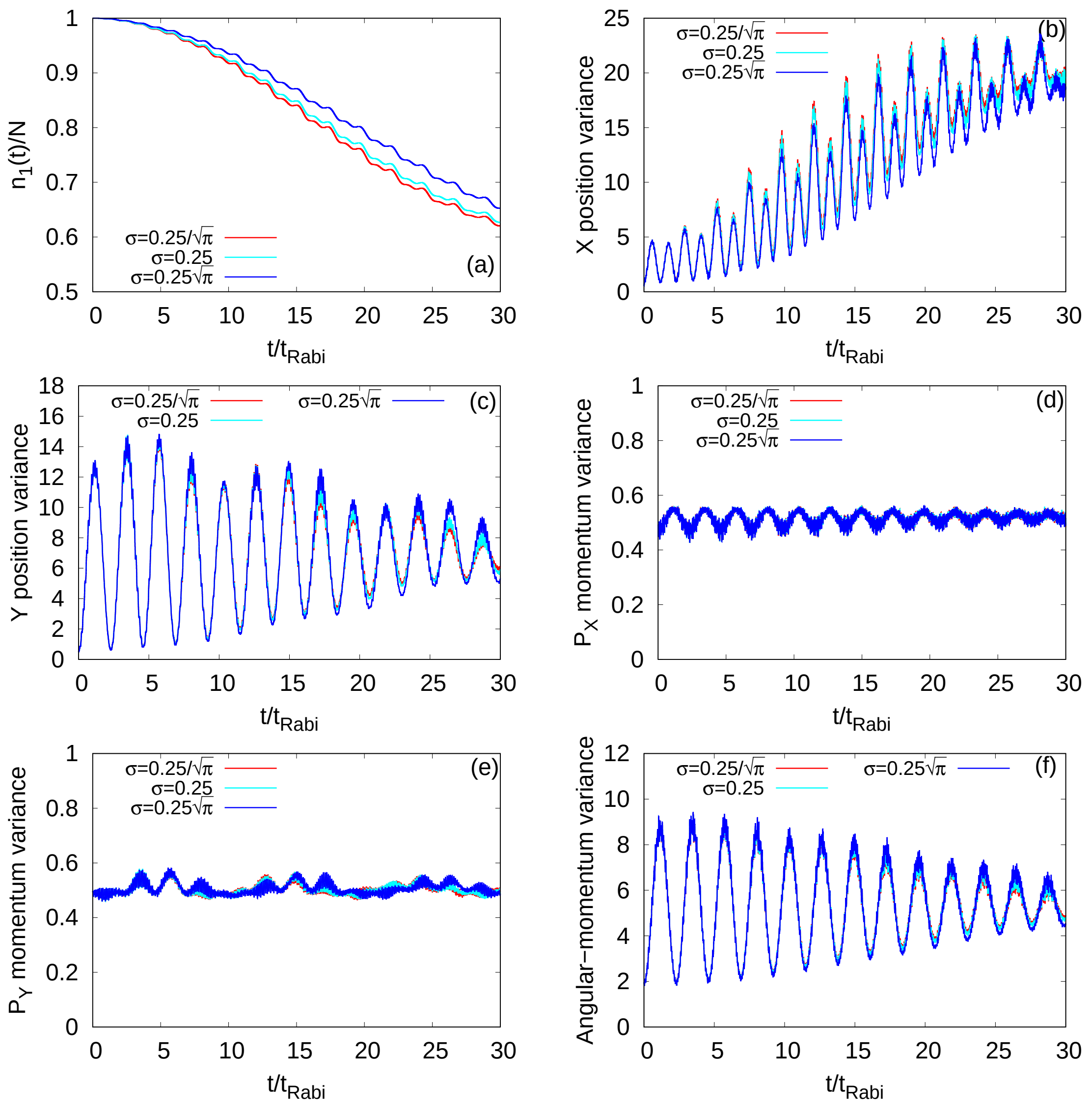

FIG. B3. Dependency of the many-body dynamics of ground state at the transversal resonant condition, $\omega_n = 0.19$, for the three different widths of interparticle interaction potential, i.e., $\sigma = 0.25/\sqrt{\pi}$, $\sigma = 0.25$, and $\sigma = 0.25\sqrt{\pi}$. The dynamics is shown by (a) $\frac{n_1(t)}{N}$, (b) $\frac{1}{N}\Delta^2_{\hat{X}}(t)$, (c) $\frac{1}{N}\Delta^2_{\hat{Y}}(t)$ (d) $\frac{1}{N}\Delta^2_{\hat{P}_X}(t)$, (e) $\frac{1}{N}\Delta^2_{\hat{P}_Y}(t)$, and (f) $\frac{1}{N}\Delta^2_{\hat{L}_Z}(t)$. The dynamics are computed with $N = 10$ bosons and the interaction parameter $\Lambda = 0.01\pi$. The many-body dynamics are computed with $M = 6$ time-dependent orbitals. The quantitative many-body results are found to be weakly dependent on σ. We show here dimensionless quantities. Color codes are explained in each panel.

[1] Alon, O. E. Analysis of a Trapped Bose-Einstein Condensate in Terms of Position, Momentum, and Angular-Momentum Variance. Symmetry **11**, 1344 (2019).

[2] Lode, A. U. J., Lévêque, C., Madsen, L. B., Streltsov, A. I. & Alon, O. E. Colloquium: Multiconfigurational time-dependent Hartree approaches for indistinguishable particles. Rev. Mod. Phys. **92**, 011001 (2020).
