# Supplementary material for: Longitudinal and transversal resonant tunneling of interacting bosons in a two-dimensional Josephson junction
Source: Sci Rep. 2022 Jan 12;12:627. doi: 10.1038/s41598-021-04312-6 (PMC8755734; doi:10.1038/s41598-021-04312-6)
Supplement: Supplementary file 2 — Supplementary Information 2. [file 41598_2021_4312_MOESM2_ESM.pdf]

Supplemental material for Longitudinal and transversal resonant tunneling of interacting bosons in a two-dimensional Josephson junction

Anal Bhowmik[1, 2, *] and Ofir E. Alon[1, 2]

[1]*Department of Mathematics, University of Haifa, Haifa 3498838, Israel*

[2]*Haifa Research Center for Theoretical Physics and Astrophysics, University of Haifa, Haifa 3498838, Israel*

In this supplemental material, we start with the investigation of the mean-field and many-body dynamics of some physical quantities, namely, position variance per particle along the x-direction, $\frac{1}{N}\Delta^2_{\hat{X}}(t)$, momentum variance per particle along the x-direction, $\frac{1}{N}\Delta^2_{\hat{P}_X}(t)$, and variance per particle of the z- component of the angular-momentum operator, $\frac{1}{N}\Delta^2_{\hat{L}_Z}(t)$, which are mostly affected by the many-body correlations in a symmetric 2D double-well potential. Further, we provide the convergences of the quantities discussed in the main text along with further complementary results and their analysis. The convergences of the results are shown with respect to the number of time-adaptive orbitals and discrete-variable-representation grid points. The results are calculated by applying multiconfigurational time-dependent Hartree for bosons (MCTDHB) method [1–3] and for our numerical computations, we use the numerical implementation in [4, 5]. We demonstrate the details of the fragmentation in terms of the occupancy of the higher natural orbitals for both resonant tunneling conditions which were not discussed in the main text. Moreover, we graphically display the position and momentum variances [6, 7] along the y-direction for the longitudinal resonant tunneling at the mean-field and many-body levels (not shown in the main text). We have shown in the main text that for Ψ_G and Ψ_X, the transversal resonant conditions are satisfied at $\omega_n = 0.19$ and 0.18, respectively, and demonstrated the variances only at the resonant values of ω_n. Here, we present all the many-body variances [8, 9], discussed in the main text, along with their convergences at $\omega_n = 0.18$, 0.19, and 0.20 for both the initial states. $M = 1$ represents the mean-field level. Here, the results are displayed for $M = 6$ and $M = 10$ time-adaptive orbitals for Ψ_G and Ψ_X, and $M = 10$ and $M = 12$ time-adaptive orbitals for Ψ_Y. The fully converged results imply that $M = 6$ time-adaptive orbitals are required to accurately present the dynamics of Ψ_G and Ψ_X in asymmetric 2D double-well potential, while for Ψ_Y, one requires $M = 10$ time-adaptive orbitals for the time considered in this work [10]. It has been checked that the size of the 2D box considered in this work is adequate for both longitudinal and transversal scenarios. Section-I and

* abhowmik@campus.haifa.ac.il

section-II describe the longitudinal and transversal resonant tunneling scenarios, respectively.

I. MEAN-FIELD AND MANY-BODY VARIANCES IN A SYMMETRIC DOUBLE-WELL POTENTIAL

We start with the graphical demonstration of the mean-field and many-body dynamics of $\frac{1}{N}\Delta^2_{\hat{X}}(t)$, $\frac{1}{N}\Delta^2_{\hat{P}_X}(t)$, and $\frac{1}{N}\Delta^2_{\hat{L}_Z}(t)$ for the initial states Ψ_G, Ψ_Y, and Ψ_X in the symmetric 2D double-well potential. As discussed in the main text, the symmetric double-well potential can be achieved by choosing either $c = 0$ in Eqn. (4) or $\omega_n = 1$ in Eqn. (6) of the main text. The results in the 2D symmetric double-well potential serve as a reference for the dynamics occurred in the resonant tunneling scenarios. Fig. S1 exhibits that all the quantities vary in time in an oscillatory manner. Also, the mean-field and many-body dynamics slowly deviate from each other with the loss of coherence of the initial states. The rate of deviation of a particular physical quantity depends on the choice of initial state and the development of the longitudinal and transversal excitations.

The mean-field and many-body dynamics of $\frac{1}{N}\Delta^2_{\hat{X}}(t)$, shown in Fig. S1 (a), (b), and (c), display oscillatory motion for all the initial states with the highest frequency of oscillations for Ψ_X. Due to the growing degree of quantum correlations, the many-body dynamics of $\frac{1}{N}\Delta^2_{\hat{X}}(t)$ show a deviation with respect to its mean-field dynamics. The maximal deviation occurs for Ψ_G till the time considered here. Further, we notice high-frequency small-amplitude oscillations on top of the peaks of the large-amplitude oscillations of $\frac{1}{N}\Delta^2_{\hat{X}}(t)$.

In the symmetric 2D double-well potential, the mean-field dynamics of $\frac{1}{N}\Delta^2_{\hat{P}_X}(t)$ for the initial states Ψ_G, Ψ_Y, and Ψ_X oscillates around the values 0.5, 0.5, and 1.5, respectively, but the many-body $\frac{1}{N}\Delta^2_{\hat{P}_X}(t)$ shows oscillations with a slowly growing values with the maximal deviation occurs for Ψ_X with respect to the corresponding mean-field dynamics. Moreover, we notice the prominent high frequency oscillations occurring in the dynamics of $\frac{1}{N}\Delta^2_{\hat{P}_X}(t)$ for the state Ψ_X.

In the dynamical evolution of $\frac{1}{N}\Delta^2_{\hat{L}_Z}(t)$, we observe a marginal difference calculated at the mean-field and many-body levels for the states Ψ_G and Ψ_X with a larger amplitude of oscillations for Ψ_X. Unlike Ψ_G and Ψ_X, Ψ_Y display a completely different dynamical behavior for $\frac{1}{N}\Delta^2_{\hat{L}_Z}(t)$ at the many-body level compared to its mean-field results. Therefore, Fig. S1 (h) exhibits that combining the excitation in the y-direction and fragmentation occurring due to the barrier in the x-direction, the many-body dynamics of $\frac{1}{N}\Delta^2_{\hat{L}_Z}(t)$ for the state Ψ_Y significantly deviates compared to the corresponding mean-field dynamics.

Before we complete this section, it is worthwhile to highlight that the mean-field and many-body

dynamics of the position and momentum variances along the y-direction practically overlap with almost constant values, being 0.5, 1.5, and 0.5 for the states Ψ_G, Ψ_Y, and Ψ_X, respectively [10].

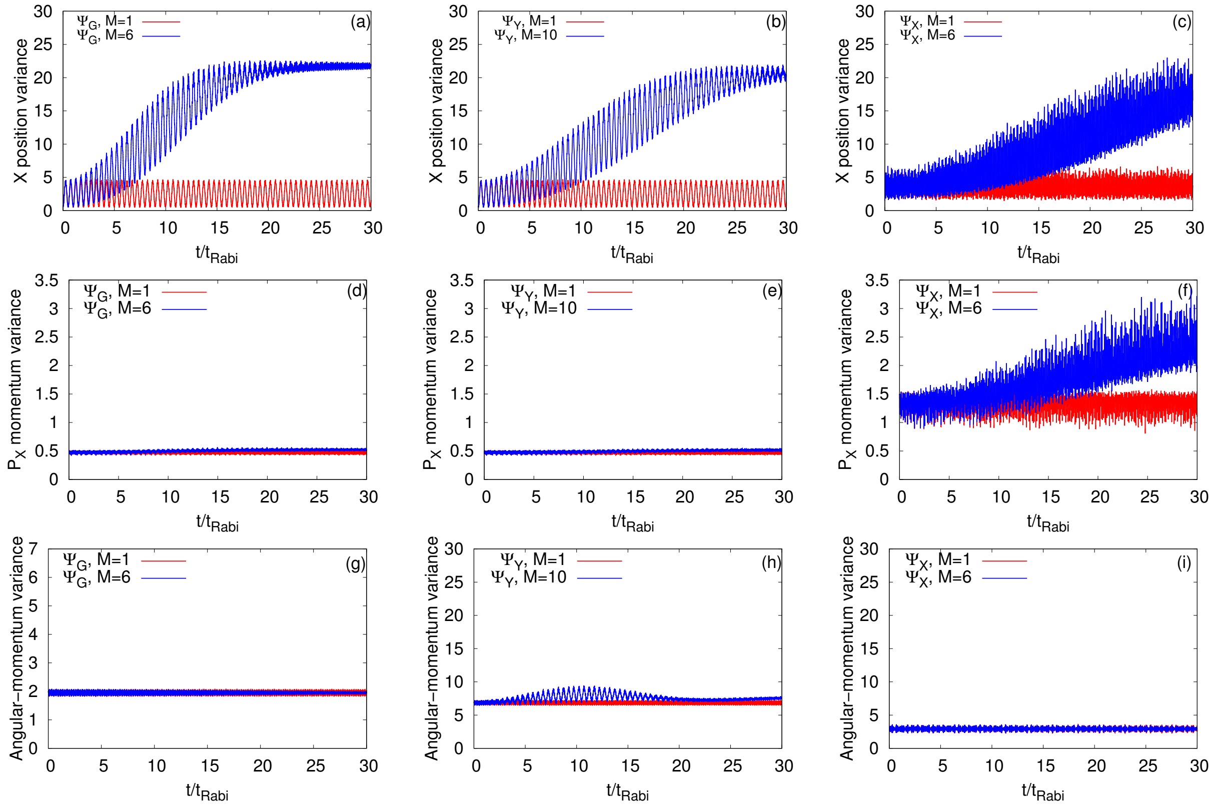

FIG. S1. Time-dependent position variance per particle along the x-direction (first row), $\frac{1}{N}\Delta^2_{\hat{X}}(t)$, momentum variance per particle along the x-direction (second row), $\frac{1}{N}\Delta^2_{\hat{P}_X}(t)$, and variance per particle of the z- component of the angular-momentum operator (third row), $\frac{1}{N}\Delta^2_{\hat{L}_Z}(t)$, in a symmetric 2D double-well potential for Ψ_G (first column), Ψ_Y (second column), and Ψ_X (third column) of $N = 10$ bosons with the interaction parameter $\Lambda = 0.01\pi$. The symmetric double-well potential can be achieved either by selecting the asymmetry parameter $c = 0$ in a longitudinally-asymmetric double-well potential or $\omega_n = 1$ in transversely-asymmetric double-well potential. $M = 1$ signifies the mean-field results. The many-body dynamics are computed with $M = 6$ time-dependent orbitals for Ψ_G and Ψ_X, and $M = 10$ time-dependent orbitals for Ψ_Y. We show here dimensionless quantities. Color codes are explained in each panel.

II. CONVERGENCES OF QUANTITIES IN LONGITUDINAL RESONANT TUNNELING

Here, the convergences of the many-body quantities in the longitudinal resonant tunneling condition are illustrated for the ground (Ψ_G) and transversely-excited (Ψ_Y) states. The computations

performed in the main text with $M = 6$ and 10 time-adaptive orbitals for Ψ_G and Ψ_Y, respectively. Here, to testify the convergence with the orbital numbers, we repeat our computations with $M = 10$ and 12 time-adaptive orbitals for Ψ_G and Ψ_Y, respectively. The bosonic clouds have the number of bosons $N = 10$ and interaction parameter $\Lambda = 0.01\pi$. Also, the many-body Hamiltonian is represented by 64×64 exponential discrete-variable-representation grid points in a box size $[-10, 10) \times [-10, 10)$. Here, we demonstrate the numerical convergences for the loss of coherence, fragmentation, and the variances of position, momentum, and angular-momentum.

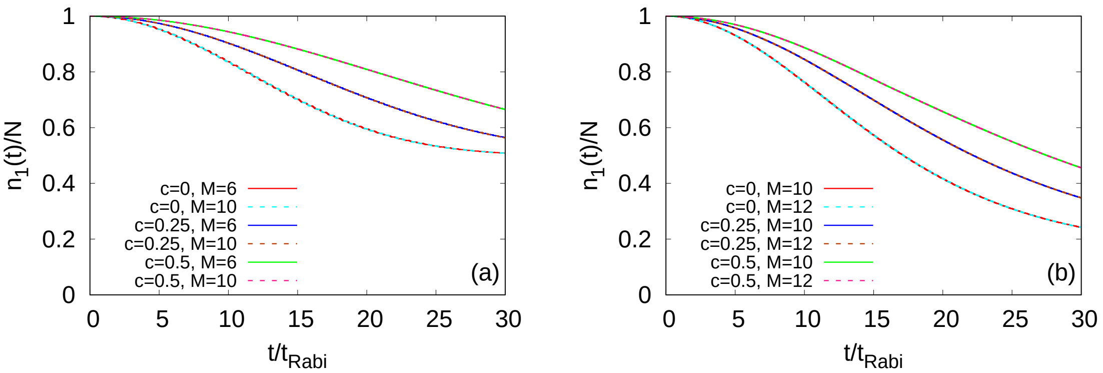

FIG. S2. Convergence of the occupation numbers per particle of the first natural orbital, $\frac{n_1(t)}{N}$, with the number of time-adaptive orbitals for the initial states (a) Ψ_G and (b) Ψ_Y in longitudinally-asymmetric 2D double-well potential. The number of interacting bosons is $N = 10$ and the interaction parameter $\Lambda = 0.01\pi$. The asymmetry parameters are $c = 0$, 0.25, and 0.5. The many-body results are computed using the MCTDHB method. The convergences is verified with $M = 6$, 10 time-adaptive orbitals for the state Ψ_G. While we demonstrate the convergence of the results for Ψ_Y using $M = 10$, 12 time-adaptive orbitals. See the text for more details. The quantities shown are dimensionless. Color codes are explained in each panel.

Fig. S2 represents the convergence of a basic quantity, loss of coherence, of the bosonic clouds, Ψ_G and Ψ_Y, in terms of the occupation numbers per particle of the first natural orbitals, $\frac{n_1(t)}{N}$. The figure showcases the results for all the asymmetry parameters, i.e., $c = 0$, 0.25, and 0.5, discussed in the main text. For both the states, $\frac{n_1(t)}{N}$ decays with time with a background fluctuations due to the growing degree of quantum correlations. The plots show that $\frac{n_1(t)}{N}$ computed from a larger number of time-adaptive orbitals falls on top of the corresponding results calculated using a smaller number of time-adaptive orbitals. The overlapping of the curves exhibits the convergence of $\frac{n_1(t)}{N}$ with the number of orbitals.

Having analyzed the convergence of $\frac{n_1(t)}{N}$, we investigate the further details of how the coherence is lost in the system by examining the development of fragmentation. Fig. S3 displays the time

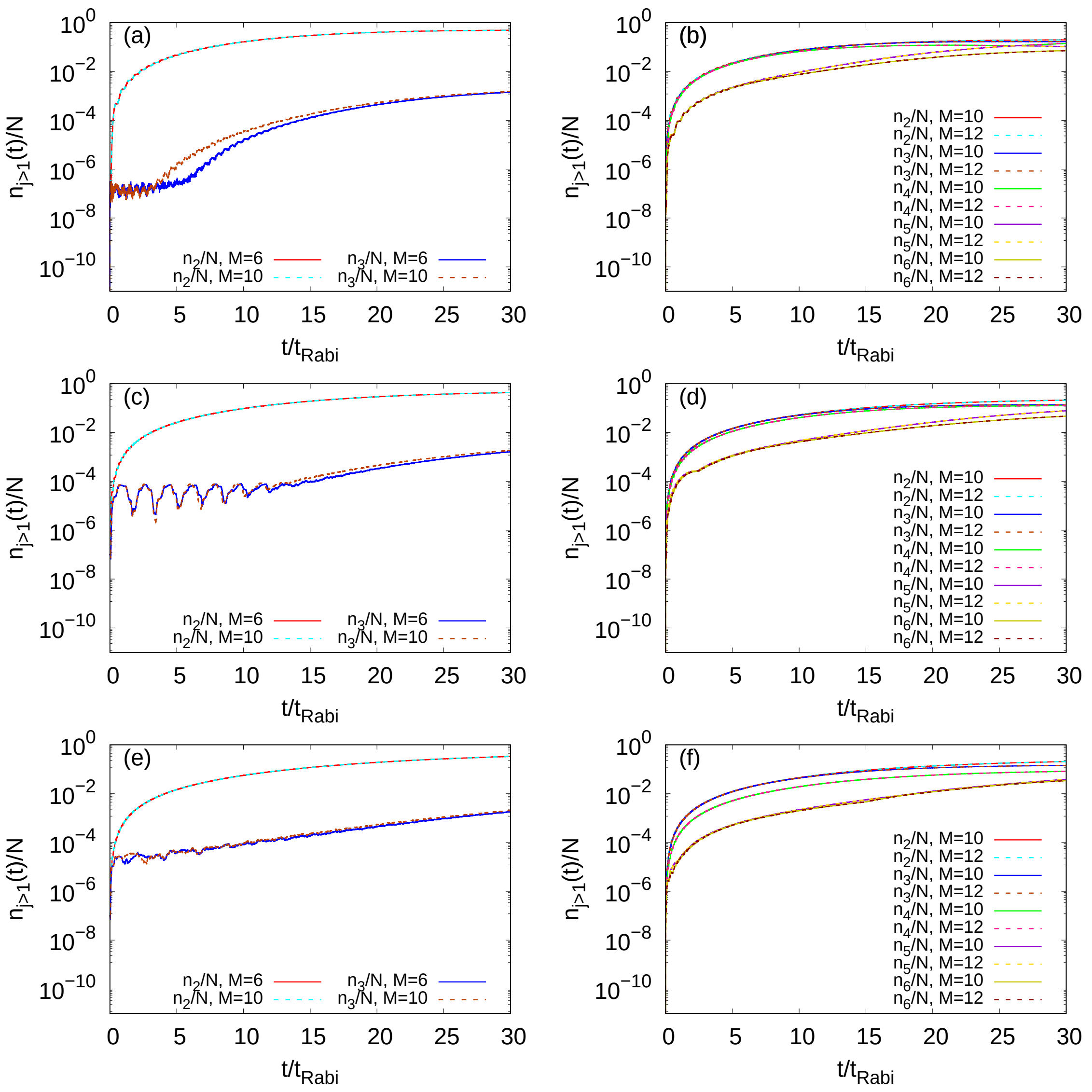

FIG. S3. Convergence of the natural occupation numbers per particle, $\frac{n_{j>1}(t)}{N}$, with the number of time-adaptive orbitals for the initial states Ψ_G (left column) and Ψ_Y (right column) in longitudinally-asymmetric 2D double-well potential. The number of interacting bosons is $N = 10$ and the interaction parameter $\Lambda = 0.01\pi$. The results for the asymmetry parameters $c = 0$, 0.25, and 0.5 are presented row-wise. The many-body results are computed using the MCTDHB method. The convergence is verified with $M = 6$, 10 time-adaptive orbitals for the state Ψ_G. While we demonstrate the convergence of the results for Ψ_Y using $M = 10$, 12 time-adaptive orbitals. See the text for more details. The quantities shown are dimensionless. Color codes are explained in each panel.

evolution of the occupancies of the higher natural orbitals per particle, $\frac{n_{j>1}(t)}{N}$, for Ψ_G and Ψ_Y with their convergences with the number of time-adaptive orbitals. As presented in the discussion of loss of coherence, here also, we plot $\frac{n_{j>1}(t)}{N}$ for the asymmetry parameters, $c = 0$, 0.25, and 0.5.

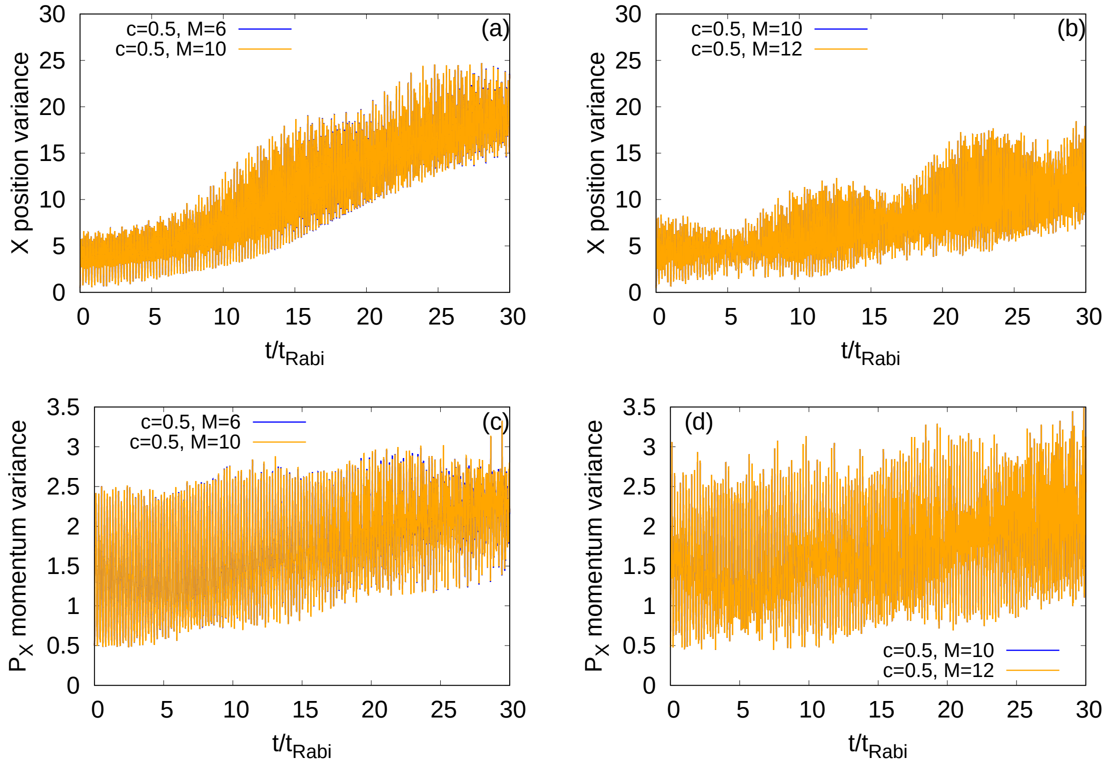

FIG. S4. Convergences of the time-dependent many-body position (1st row) and momentum (2nd row) variances per particle along the x-direction, $\frac{1}{N}\Delta^2_{\hat{X}}(t)$ and $\frac{1}{N}\Delta^2_{\hat{P}_X}(t)$, respectively, with the number of time-adaptive orbitals for the initial states Ψ_G (left column) and Ψ_Y (right column) in longitudinally-asymmetric 2D double-well potential. The number of interacting bosons is $N = 10$ and the interaction parameter $\Lambda = 0.01\pi$. The results are for the asymmetry parameter $c = 0.5$. The many-body results are computed using the MCTDHB method. The convergence is verified with $M = 6$, 10 time-adaptive orbitals for the state Ψ_G. While we demonstrate the convergence of the results for Ψ_Y using $M = 10$, 12 time-adaptive orbitals. See the text for more details. The quantities shown are dimensionless. Color codes are explained in each panel.

The higher natural orbitals which have significant amount of occupancies for Ψ_G (second and third natural orbitals) and Ψ_Y (second to sixth natural orbitals) are shown in the figure for all asymmetry parameters. To demonstrate the convergence with the time-adaptive orbital numbers, we present the fragmentation dynamics with $M = 6$, 10 time-adaptive orbitals for Ψ_G and $M = 10$, 12 time-adaptive orbitals for Ψ_Y. Here it is observed that as time passes by the occupations of all the higher natural orbitals gradually increase. Moreover, $\frac{n_3(t)}{N}$ of Ψ_G are oscillatory first and then increasing, clearly visible at $c = 0.25$. We notice that only two higher natural orbitals are considerably occupied for Ψ_G but for Ψ_Y, the amount of occupancies of the five higher natural orbitals are rather significant. The comparison of the fragmentation dynamics of Ψ_G and Ψ_Y exhibits that

whenever the transverse excitations exist in the system, more natural orbitals are required to accurately capture the dynamical behavior. However, the occupancies of the higher natural orbitals computed from a larger number of time-adaptive orbitals overlap with the respective results found from a smaller number of time-adaptive orbitals, indicating the convergence of the fragmentation dynamics for Ψ_G and Ψ_Y. The convergences of the occupancies of all the orbitals demonstrated till now automatically imply the convergence of the most basic quantity, i.e., survival probability, see Fig. 3 in the main text.

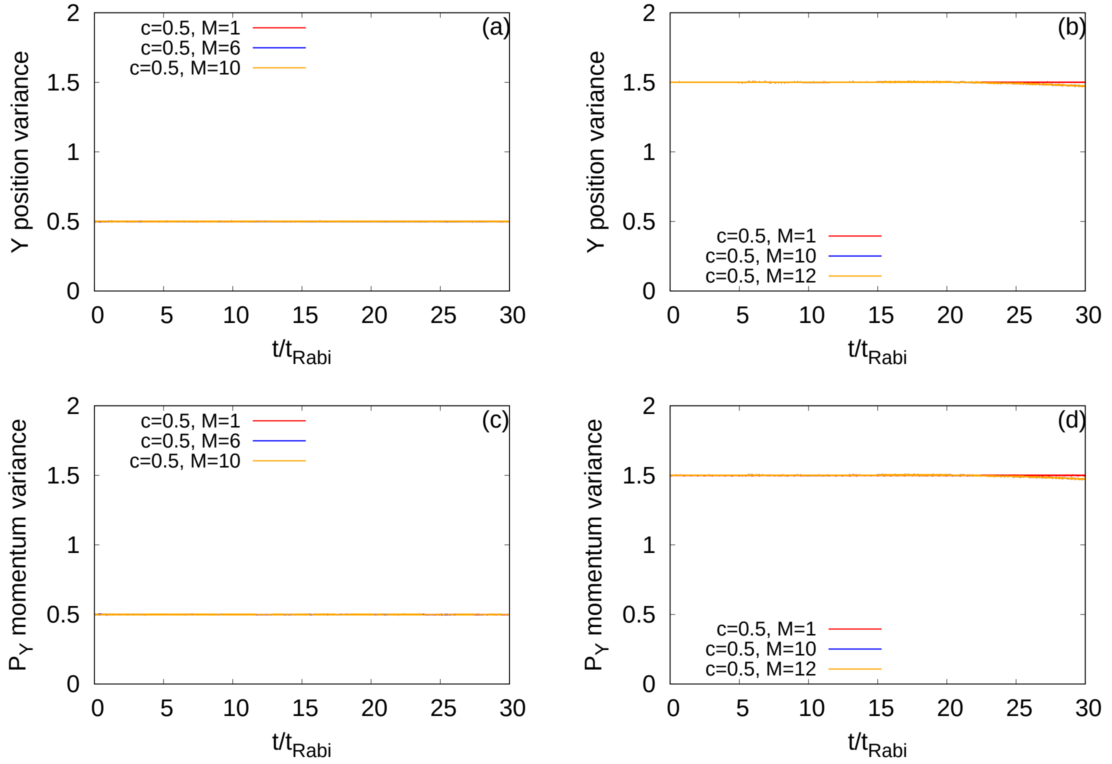

FIG. S5. Convergences of the time-dependent many-body position (1st row) and momentum (2nd row) variances per particle along the y-direction, $\frac{1}{N}\Delta^2_{\hat{Y}}(t)$ and $\frac{1}{N}\Delta^2_{\hat{P}_Y}(t)$, respectively, with the number of time-adaptive orbitals for the initial states Ψ_G (left column) and Ψ_Y (right column) in longitudinally-asymmetric 2D double-well potential. The number of interacting bosons is $N = 10$ and the interaction parameter $\Lambda = 0.01\pi$. The results are for the asymmetry parameter $c = 0.5$. The many-body results are computed using the MCTDHB method. $M = 1$ shows the mean-field results. The convergence is verified with $M = 6$, 10 time-adaptive orbitals for the state Ψ_G. While we demonstrate the convergence of the results for Ψ_Y using $M = 10$, 12 time-adaptive orbitals. See the text for more details. The quantities shown are dimensionless. Color codes are explained in each panel.

The time evolution of the many-body position and momentum variances per particle along the x-direction, $\frac{1}{N}\Delta^2_{\hat{X}}(t)$ and $\frac{1}{N}\Delta^2_{\hat{P}_X}(t)$, respectively, along with their numerical convergences

with respect to the orbital numbers are illustrated in Fig. S4. Here, we checked and verified the convergences of $\frac{1}{N}\Delta^2_{\hat{X}}(t)$ and $\frac{1}{N}\Delta^2_{\hat{P}_X}(t)$ for all the asymmetry parameters discussed in the main text. But, to demonstrate the convergence, we choose only the second resonant tunneling condition, i.e., $c = 0.5$. In the main text, we discussed that, at $c = 0.5$, $\frac{1}{N}\Delta^2_{\hat{X}}(t)$ and $\frac{1}{N}\Delta^2_{\hat{P}_X}(t)$ have two kinds of oscillation, namely, small frequency with large amplitude (due to the density oscillation) and high frequency with small amplitude (due to the breathing mode oscillation). The panels in Fig. S4 show that both the variances are well converged with the orbital numbers. Even the oscillation which has high frequency with small amplitude, found with lower orbital numbers, overlaps with the corresponding results calculated from higher orbital numbers.

As discussed in the main text, the many-particle position and momentum variances per particle along the y-direction, $\frac{1}{N}\Delta^2_{\hat{Y}}(t)$ and $\frac{1}{N}\Delta^2_{\hat{P}_Y}(t)$, respectively, have very small fluctuations, of the order of 10^{-3} for all the asymmetry parameters discussed in this work. Here we graphically show the time evolution of $\frac{1}{N}\Delta^2_{\hat{Y}}(t)$ and $\frac{1}{N}\Delta^2_{\hat{P}_Y}(t)$ both at the mean-field and many-body levels along with their many-body convergences at $c = 0.5$, see Fig. S5. We observe that the almost frozen $\frac{1}{N}\Delta^2_{\hat{Y}}(t)$ and $\frac{1}{N}\Delta^2_{\hat{P}_Y}(t)$ for both the states, Ψ_G and Ψ_Y, are fully converged with the number of orbitals.

Fig. S6 depicts the convergence of the many-body angular-momentum variance per particle, $\frac{1}{N}\Delta^2_{\hat{L}_Z}(t)$ for both the initial states, Ψ_G and Ψ_Y. As mentioned in the dynamics of $\frac{1}{N}\Delta^2_{\hat{X}}(t)$ and $\frac{1}{N}\Delta^2_{\hat{P}_X}(t)$ at $c = 0.5$, here also, we observe the combined effect of the density and breathing mode oscillations. There is no visible difference of $\frac{1}{N}\Delta^2_{\hat{L}_Z}(t)$ found when computed from the smaller and larger numbers of orbitals, which corresponds to the convergence of $\frac{1}{N}\Delta^2_{\hat{L}_Z}(t)$ with the number of orbitals.

Now, we demonstrate the convergence of the results described in the main text with the number of grid points. So far all the results for the longitudinal resonant tunneling scenario computed with 64×64 grid points. To check the convergence with the grid points, we recomputed all the quantities with 128×128 grid points for all asymmetry parameters. In order to display the convergence of our results, we choose the most sensitive quantity, $\frac{1}{N}\Delta^2_{\hat{L}_Z}(t)$ presented in this work. The overlapping curves of $\frac{1}{N}\Delta^2_{\hat{L}_Z}(t)$ with increasing the grid density signifies that the results are fully converged for 64×64 grid points for all values of c for Ψ_G, and at $c = 0$ and 0.25 for Ψ_Y. The small difference in the results for the excited state in Fig. S7 (f) represents that $\frac{1}{N}\Delta^2_{\hat{L}_Z}(t)$ is well converged with 64×64 grid points at $c = 0.5$.

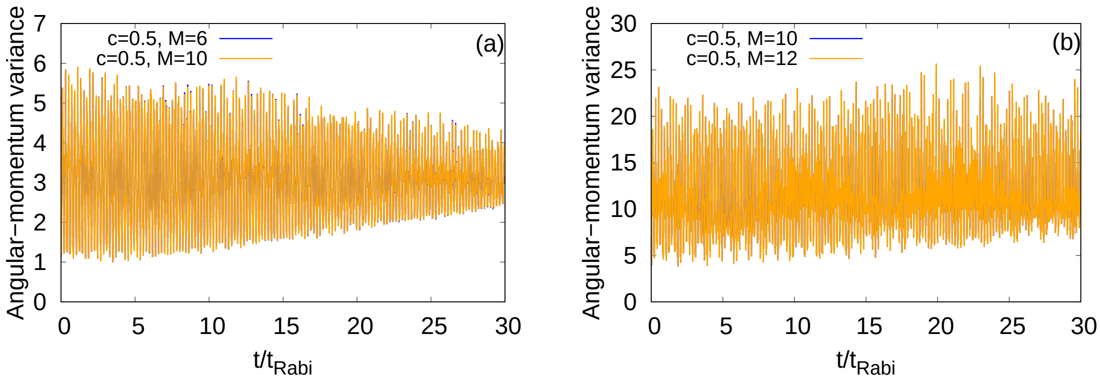

FIG. S6. Convergence of the time-dependent many-body angular-momentum variance per particle, $\frac{1}{N}\Delta^2_{\hat{L}_Z}(t)$, with the number of time-adaptive orbitals for the initial states (a) Ψ_G and (b) Ψ_Y in longitudinally-asymmetric 2D double-well potential. The number of interacting bosons is $N = 10$ and the interaction parameter $\Lambda = 0.01\pi$. The results are for the asymmetry parameter $c = 0.5$. The many-body results are computed using the MCTDHB method. The convergence is verified with $M = 6$, 10 time-adaptive orbitals for the state Ψ_G. While we demonstrate the convergence of the results for Ψ_Y using $M = 10$, 12 time-adaptive orbitals. See the text for more details. The quantities shown are dimensionless. Color codes are explained in each panel.

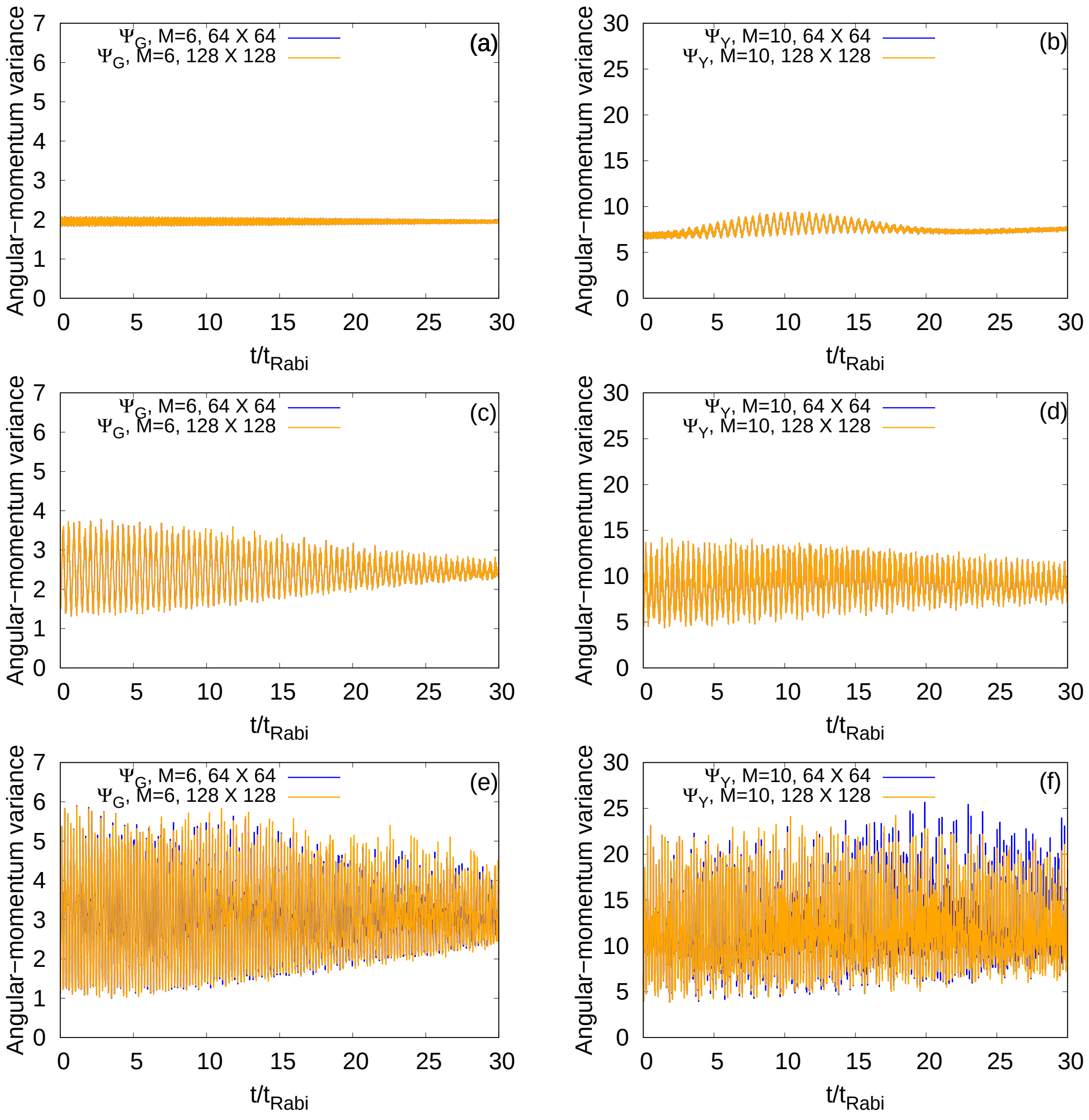

FIG. S7. Convergence of the time-dependent many-body angular-momentum variance per particle, $\frac{1}{N}\Delta^2_{\hat{L}_Z}(t)$, with the number of grid points for the initial states Ψ_G (left column) and Ψ_Y (right column) in longitudinally-asymmetric 2D double-well potential. The number of interacting bosons is $N = 10$ and the interaction parameter $\Lambda = 0.01\pi$. The results for the asymmetry parameters $c = 0$, 0.25, and 0.5 are presented row-wise. The many-body results are computed using the MCTDHB method. The convergence is verified with 64×64 and 128×128 grid points. See the text for more details. The quantities shown are dimensionless. Color codes are explained in each panel.

III. CONVERGENCES OF QUANTITIES IN TRANSVERSAL RESONANT TUNNELING

Similar to the longitudinal resonant tunneling, now we demonstrate the convergence of loss of coherence, fragmentation, and the variances of position, momentum, and angular-momentum with respect to the number of time-adaptive orbitals and the density of the discrete-variable-representation grid points. Here the initial states are Ψ_G and the longitudinally-excited state Ψ_X. In the main text, all the physical quantities in the transversal resonant tunneling for Ψ_G and Ψ_X are computed using $M = 6$ time-adaptive orbitals. To verify the convergence with respect to the number of orbitals, we recomputed all the quantities with $M = 10$ time-adaptive orbitals. For the transversal resonant tunneling, the many-body Hamiltonian is represented by 128×128 exponential discrete-variable-representation grid points in a box size of $[-10, 10) \times [-10, 10)$.

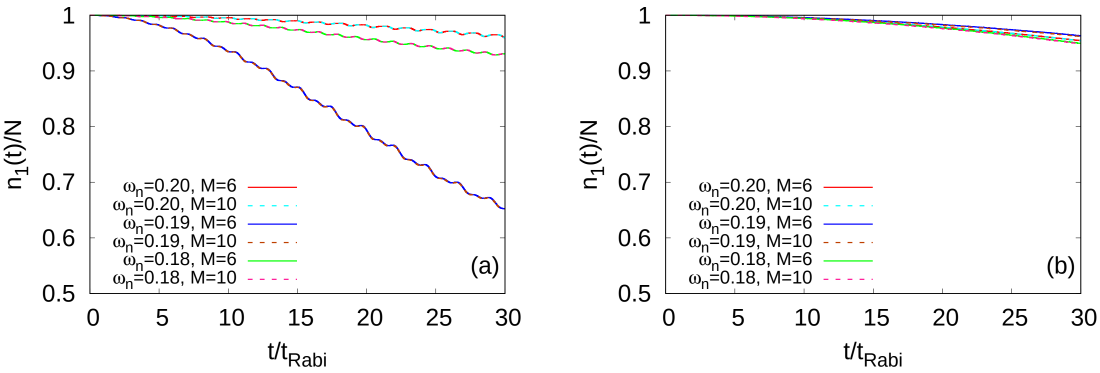

FIG. S8. Convergence of the occupation numbers per particle of the first natural orbital, $\frac{n_1(t)}{N}$, with the number of time-adaptive orbitals for the initial states (a) Ψ_G and (b) Ψ_X in transversely-asymmetric 2D double-well potential. The number of interacting bosons is $N = 10$ and the interaction parameter $\Lambda = 0.01\pi$. The frequencies are $\omega_n = 0.20$, 0.19, and 0.18. The many-body results are computed using the MCTDHB method. The convergence is verified with $M = 6$ and $M = 10$ time-adaptive orbitals for both states. See the text for more details. The quantities shown are dimensionless. Color codes are explained in each panel.

Fig. S8 depicts the numerical convergence of loss of coherence, represented as the occupation numbers per particle of the first natural orbital, $\frac{n_1(t)}{N}$, for Ψ_G and Ψ_X with the number of orbitals. The frequency of the wider right well ω_n, see Eq.(2.5) of the main text, is considered as $\omega_n = 0.20$, 0.19, and 0.18. It is noted that at $\omega_n = 0.19$ and 0.18, we find the transeversal resonant tunneling scenario for Ψ_G and Ψ_X, respectively. As discussed in the main text for $M = 6$ orbitals, here also for $M = 10$ orbitals, $\frac{n_1(t)}{N}$ decays with an oscillatory background for both initial states and falls on top of the corresponding results found from $M = 6$ orbitals, indicating the convergence of $\frac{n_1(t)}{N}$

with the number of orbitals.

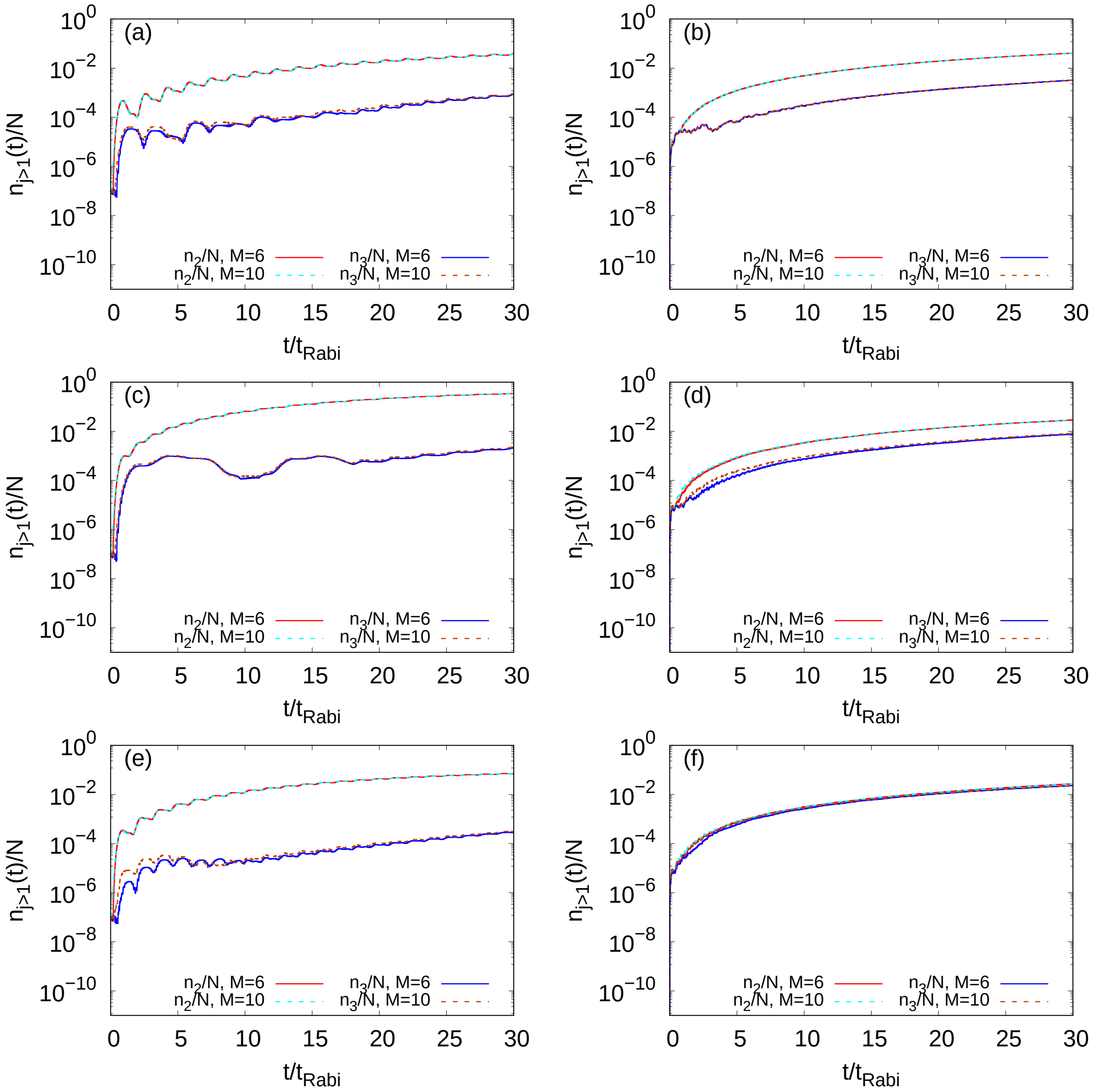

FIG. S9. Convergence of the natural occupation numbers per particle, $\frac{n_{j>1}(t)}{N}$, with the number of time-adaptive orbitals for the initial states Ψ_G (left column) and Ψ_X (right column) in transversely-asymmetric 2D double-well potential. The number of interacting bosons is $N = 10$ and the interaction parameter $\Lambda = 0.01\pi$. The results for the frequencies $\omega_n = 0.20$, 0.19, and 0.18 are presented row-wise. The many-body results are computed using the MCTDHB method. The convergence is verified with $M = 6$ and $M = 10$ time-adaptive orbitals for both states. See the text for more details. The quantities shown are dimensionless. Color codes are explained in each panel.

In order to visualize how the fragmentation develops in the transversal resonant tunneling scenario and at its vicinity, we explore the time-dependent occupancies of higher natural orbitals per particle, $\frac{n_{j>1}(t)}{N}$, along with their convergences with the number of orbitals for Ψ_G and Ψ_X.

We graphically present only $\frac{n_2(t)}{N}$ and $\frac{n_3(t)}{N}$ in Fig. S9 as they have occupations in appreciable amount. Among the three different values of ω_n, $\frac{n_2(t)}{N}$ is maximally occupied at $\omega_n = 0.19$ for Ψ_G and at $\omega_n = 0.18$ for Ψ_X, as these values of ω_n produce the transversal resonant tunneling for the respective states. We observe the oscillatory nature in the the dynamics of $\frac{n_2(t)}{N}$ and $\frac{n_3(t)}{N}$ for Ψ_G, whereas it is hardly visible for Ψ_X.

Figs. S10 and S11 show the many-body position and momentum variances per particle along the x-direction, $\frac{1}{N}\Delta^2_{\hat{X}}(t)$ and $\frac{1}{N}\Delta^2_{\hat{P}_X}(t)$, respectively, along with their numerical convergences with number of orbitals. Here we analyze the many-body $\frac{1}{N}\Delta^2_{\hat{X}}(t)$ and $\frac{1}{N}\Delta^2_{\hat{P}_X}(t)$ at $\omega_n = 0.20$, 0.19, and 0.18 for both Ψ_G and Ψ_X. $\frac{1}{N}\Delta^2_{\hat{X}}(t)$ and $\frac{1}{N}\Delta^2_{\hat{P}_X}(t)$ computed with $M = 10$ orbitals fall on top of the corresponding quantities found from $M = 6$ orbitals signifying the convergences of the quantities for both the states. For Ψ_X, we observe marginal differences in terms of frequency and amplitude for both the quantities, $\frac{1}{N}\Delta^2_{\hat{X}}(t)$ and $\frac{1}{N}\Delta^2_{\hat{P}_X}(t)$, when computed at $\omega_n = 0.20$, 0.19, and 0.18. These marginal differences are consistent with the corresponding survival probabilities discussed in the main text. The differences are negligible for both the quantities at three different frequencies for Ψ_X as it lies in the first excited band and feels a smaller barrier when tunnels. The frequency of oscillations of $\frac{1}{N}\Delta^2_{\hat{X}}(t)$ for Ψ_X is practically half of the corresponding oscillation frequecies of the survival probabilities.

Unlike Ψ_X, we find significantly different many-body dynamics of $\frac{1}{N}\Delta^2_{\hat{X}}(t)$ and $\frac{1}{N}\Delta^2_{\hat{P}_X}(t)$ for Ψ_G at $\omega_n = 0.20$, 0.19, and 0.18. In order to characterize the nature of $\frac{1}{N}\Delta^2_{\hat{X}}(t)$ for Ψ_G, we notice that the average value of $\frac{1}{N}\Delta^2_{\hat{X}}(t)$ increases as time passes by, which found maximally at the resonant value $\omega_n = 0.19$. As demonstrated for Ψ_G in the main text, the oscillation frequency of $\frac{1}{N}\Delta^2_{\hat{X}}(t)$ is twice of the oscillation frequency of the survival probability at $\omega_n = 0.19$. Dissimilar to the resonant value of ω_n, here at $\omega_n = 0.18$ and 0.20, we find that the oscillation frequencies of the survival probability and $\frac{1}{N}\Delta^2_{\hat{X}}(t)$ practically overlap with each other. At $\omega_n = 0.19$, $\frac{1}{N}\Delta^2_{\hat{X}}(t)$ displays two different amplitudes of oscillations. This signature can also be seen at $\omega_n = 0.20$ (hardly visible). The time evolution of $\frac{1}{N}\Delta^2_{\hat{P}_X}(t)$ for Ψ_G is mostly dominated by the breathing mode oscillations. As time progresses, the amplitude of the oscillation of $\frac{1}{N}\Delta^2_{\hat{P}_X}(t)$ decreases for Ψ_G at all values of ω_n considered in this work.

In Figs. S12 and S13, we display the many-body position and momentum variances per particle along the y-direction, $\frac{1}{N}\Delta^2_{\hat{Y}}(t)$ and $\frac{1}{N}\Delta^2_{\hat{P}_Y}(t)$, respectively, for Ψ_G and Ψ_X at $\omega_n = 0.20$, 0.19, and 0.18, along with their numerical convergences with the number of orbitals. For both states,

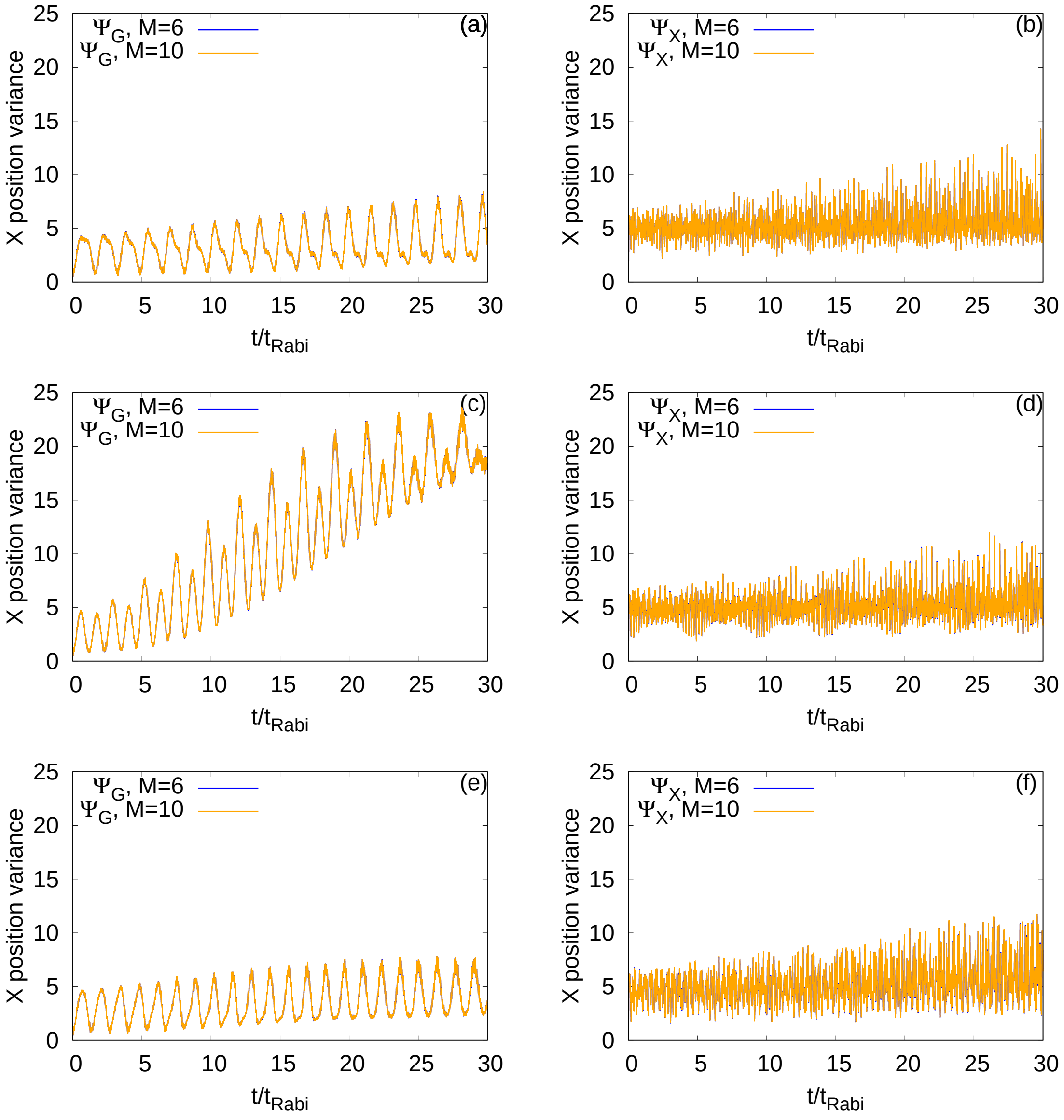

FIG. S10. Convergences of the time-dependent many-body position variance per particle along the x-direction, $\frac{1}{N}\Delta^2_{\hat{X}}(t)$, with the number of time-adaptive orbitals for the initial states Ψ_G (left column) and Ψ_X (right column) in transversely-asymmetric 2D double-well potential. The number of interacting bosons is $N = 10$ and the interaction parameter $\Lambda = 0.01\pi$. The results for the frequencies $\omega_n = 0.20$, 0.19, and 0.18 are presented row-wise. The many-body results are computed using the MCTDHB method with $M = 6$ time-adaptive orbitals. The convergence is verified with $M = 10$ time-adaptive orbitals. See the text for more details. The quantities shown are dimensionless. Color codes are explained in each panel.

$\frac{1}{N}\Delta^2_{\hat{Y}}(t)$ and $\frac{1}{N}\Delta^2_{\hat{P}_Y}(t)$ computed using $M = 6$ and $M = 10$ time-adaptive orbitals fall on top of each other signifying the convergence of the quantities with the number of orbitals. For Ψ_G, the amplitude of the many-body time evolution of $\frac{1}{N}\Delta^2_{\hat{Y}}(t)$ decays with the growing degree of

correlations, and the decay rate is maximal at $\omega_n = 0.19$ and minimal at $\omega_n = 0.20$. At the resonant value for Ψ_G, $\omega_n = 0.19$, the amplitude of $\frac{1}{N}\Delta^2_{\hat{Y}}(t)$ reaches upto around 15, while at the other frequencies they are comparatively small, i.e., around 4.5 at $\omega_n = 0.18$ and around 6.5 at $\omega_n = 0.20$. For the slower rate of growth of the fragmentation for Ψ_X, the decay rate of the many-body $\frac{1}{N}\Delta^2_{\hat{Y}}(t)$ is hardly visible even at the resonant value of ω_n, see Fig. S12. By comparing the many-body $\frac{1}{N}\Delta^2_{\hat{Y}}(t)$ at different frequencies for Ψ_X, we observe that at $\omega_n = 0.18$, the resonant condition for Ψ_X, the amplitude of oscillation reaches upto 12 whereas at $\omega_n = 0.19$ and $\omega_n = 0.20$, the respective amplitudes reach upto around 10.5 and 9, respectively.

Focusing on the momentum variance along the y-direction for Ψ_G, we notice that at $\omega_n = 0.18$ and $\omega_n = 0.20$, $\frac{1}{N}\Delta^2_{\hat{P}_Y}(t)$ have the amplitudes of fluctuations are of the order of 10^{-2} and at the resonant value the respective fluctuation of amplitude becomes 10^{-1}. Unlike Ψ_G, the amplitude of fluctuation of $\frac{1}{N}\Delta^2_{\hat{P}_Y}(t)$ for Ψ_X is comparatively large, in between 0.23 to 0.60, at all three frequencies.

Fig. S14 collects the numerical convergence of angular-momentum variance per particle, $\frac{1}{N}\Delta^2_{\hat{L}_Z}(t)$, for Ψ_G and Ψ_X at $\omega_n = 0.20$, 0.19, and 0.18. As discussed for $\frac{1}{N}\Delta^2_{\hat{Y}}(t)$, here also the amplitude of $\frac{1}{N}\Delta^2_{\hat{L}_Z}(t)$ decays with time for Ψ_G. The decay rate is the slowest at $\omega_n = 0.20$ and fastest at $\omega_n = 0.19$. But for Ψ_X, $\frac{1}{N}\Delta^2_{\hat{L}_Z}(t)$ does not follow the trend of decaying amplitude as Ψ_G. In general, we observe that at resonant value of ω_n, $\frac{1}{N}\Delta^2_{\hat{L}_Z}(t)$ takes maximum value compared to other frequencies considered here. Moreover, comparing the many-body time evolution of $\frac{1}{N}\Delta^2_{\hat{L}_Z}(t)$, computed using $M = 6$ and 10 orbitals, signifies that the results are well converged with $M = 6$ orbitals.

The main text and so far the supplemental material describe all quantities, i.e., the survival probability, loss of coherence, fragmentation, and the variances, for the transversal resonant scenario with 128×128 grid points. In order to verify the convergence with the grid points, we repeat our computation with 256×256 grid points for both objects, Ψ_G and Ψ_X. To demonstrate the convergence with the grid points, we display $\frac{1}{N}\Delta^2_{\hat{L}_Z}(t)$ which is the most sensitive quantity discussed in this work. Fig. S15 displays the convergence of the many-body $\frac{1}{N}\Delta^2_{\hat{L}_Z}(t)$ at $\omega_n = 0.19$ for Ψ_G and $\omega_n = 0.18$ for Ψ_X, as there is no visible effect with increasing the number of grid points.

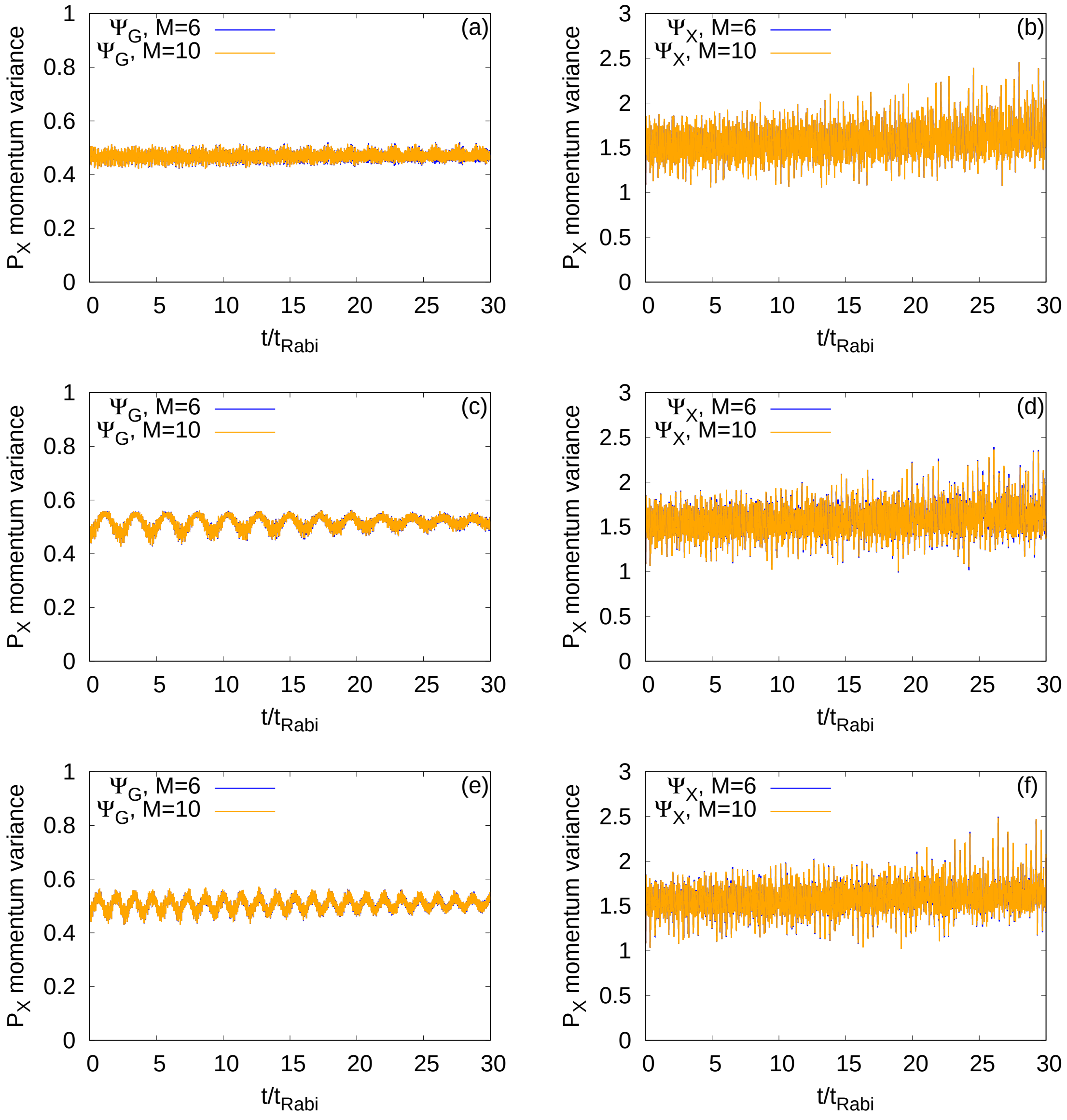

FIG. S11. Convergences of the time-dependent many-body momentum variance per particle along the x-direction, $\frac{1}{N}\Delta^2_{\hat{P}_X}(t)$, with the number of time-adaptive orbitals for the initial states Ψ_G (left column) and Ψ_X (right column) in transversely-asymmetric 2D double-well potential. The number of interacting bosons is $N = 10$ and the interaction parameter $\Lambda = 0.01\pi$. The results for the frequencies $\omega_n = 0.20$, 0.19, and 0.18 are presented row-wise. The many-body results are computed using the MCTDHB method with $M = 6$ time-adaptive orbitals. The convergence is verified with $M = 10$ time-adaptive orbitals. See the text for more details. The quantities shown are dimensionless. Color codes are explained in each panel.

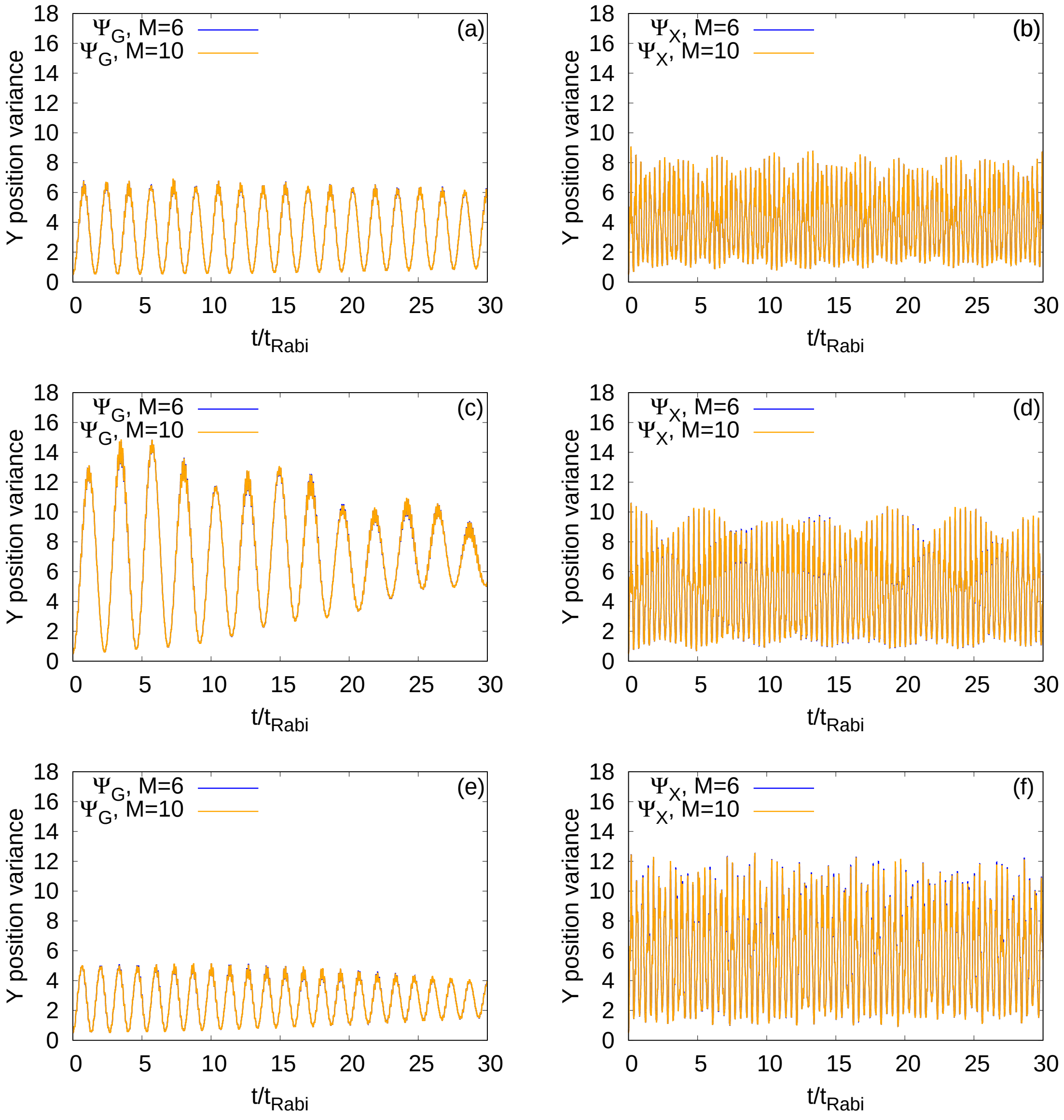

FIG. S12. Convergences of the time-dependent many-body position variance per particle along the y-direction, $\frac{1}{N}\Delta^2_{\hat{Y}}(t)$, with the number of time-adaptive orbitals for the initial states Ψ_G (left column) and Ψ_X (right column) in transversely-asymmetric 2D double-well potential. The number of interacting bosons is $N = 10$ and the interaction parameter $\Lambda = 0.01\pi$. The results for the frequencies $\omega_n = 0.20$, 0.19, and 0.18 are presented row-wise. The many-body results are computed using the MCTDHB method with $M = 6$ time-adaptive orbitals. The convergence is verified with $M = 10$ time-adaptive orbitals. See the text for more details. The quantities shown are dimensionless. Color codes are explained in each panel.

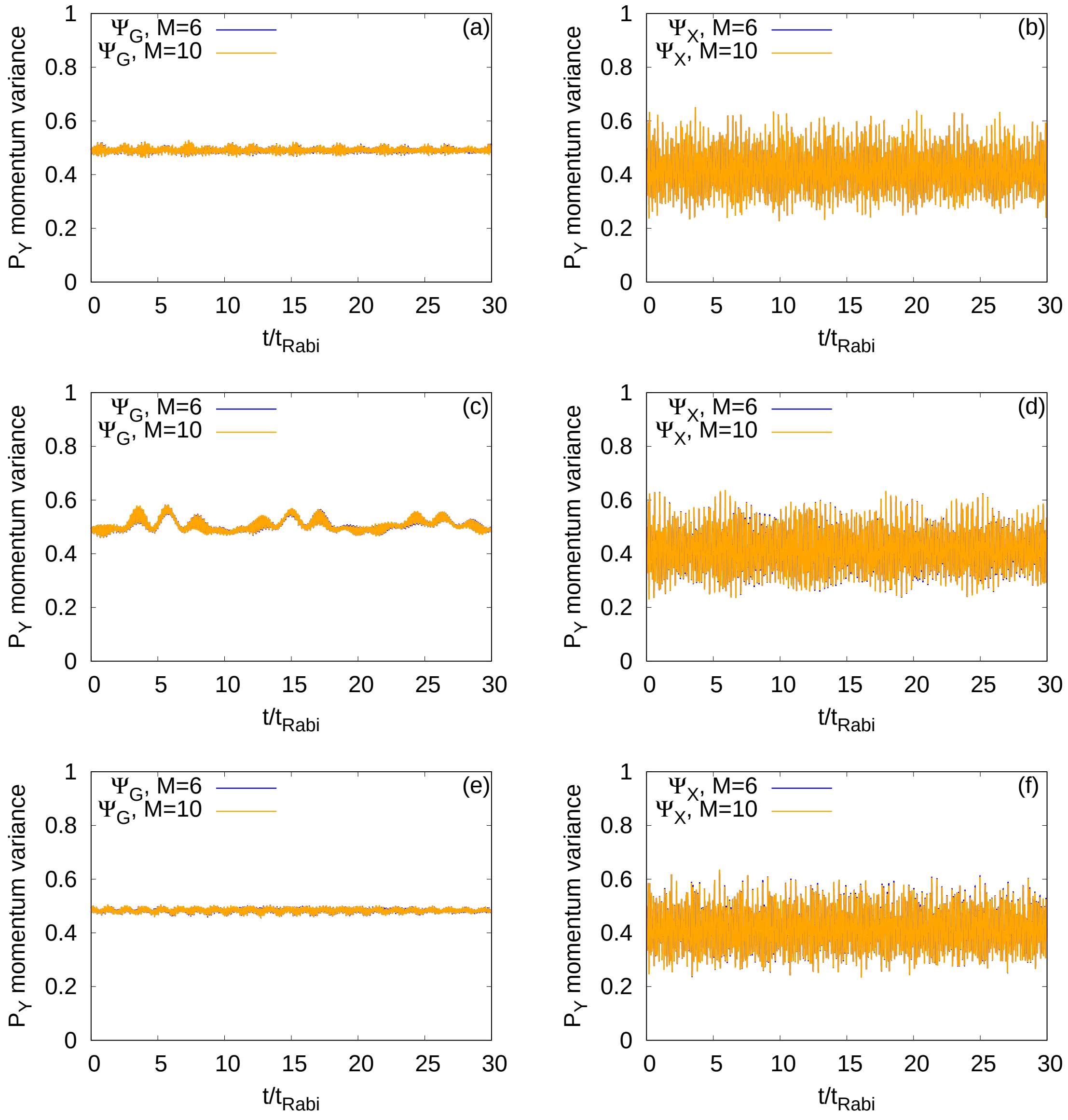

FIG. S13. Convergences of the time-dependent many-body momentum variance per particle along the y-direction, $\frac{1}{N}\Delta^2_{\hat{P}_Y}(t)$, with the number of time-adaptive orbitals for the initial states Ψ_G (left column) and Ψ_X (right column) in transversely-asymmetric 2D double-well potential. The number of interacting bosons is $N = 10$ and the interaction parameter $\Lambda = 0.01\pi$. The results for the frequencies $\omega_n = 0.20$, 0.19, and 0.18 are presented row-wise. The many-body results are computed using the MCTDHB method with $M = 6$ time-adaptive orbitals. The convergence is verified with $M = 10$ time-adaptive orbitals. See the text for more details. The quantities shown are dimensionless. Color codes are explained in each panel.

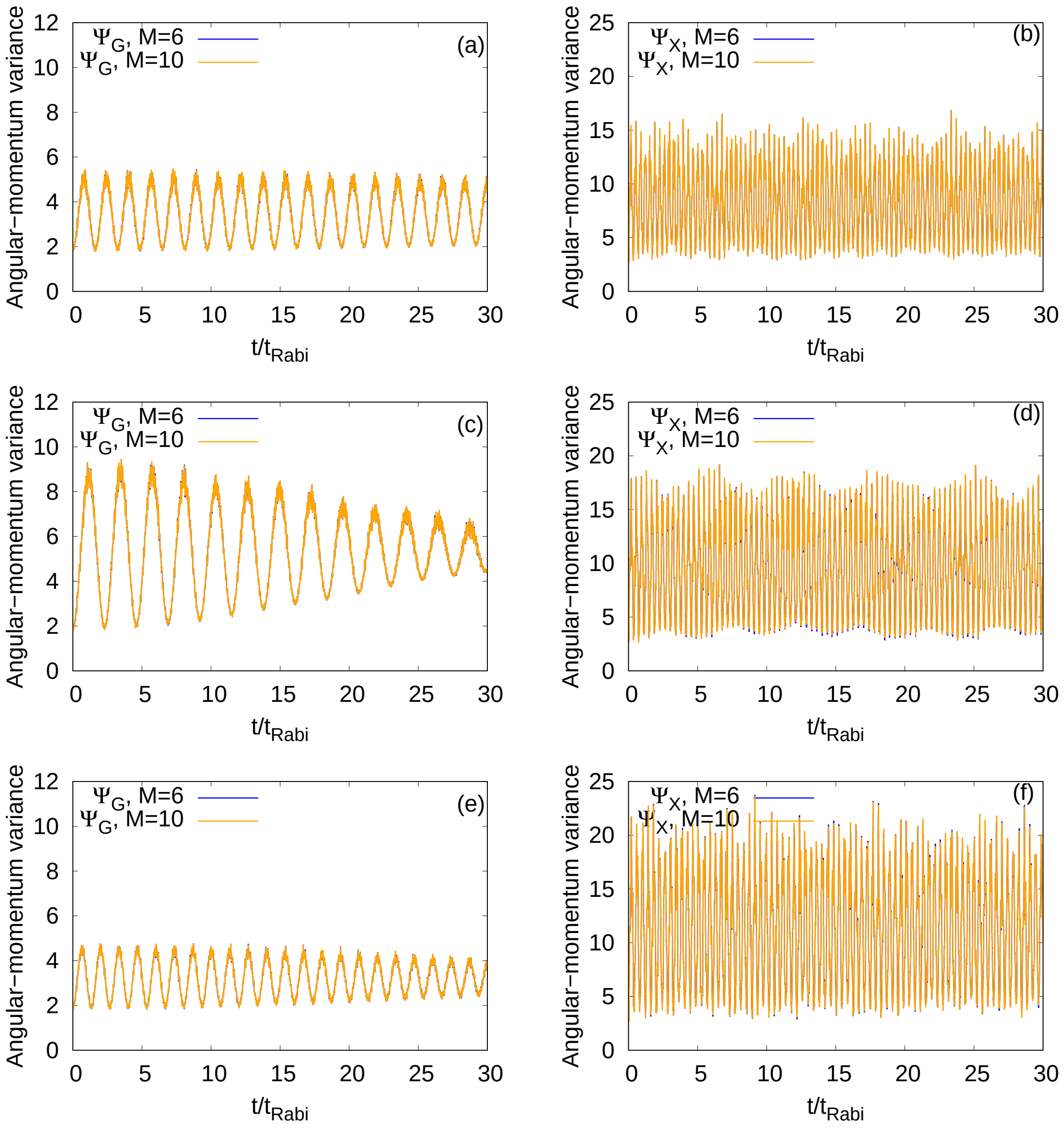

FIG. S14. Convergences of the time-dependent many-body angular-momentum variance of z- component per particle, $\frac{1}{N}\Delta^2_{\hat{L}_Z}(t)$, with the number of time-adaptive orbitals for the initial states Ψ_G (left column) and Ψ_X (right column) in transversely-asymmetric 2D double-well potential. The number of interacting bosons is $N = 10$ and the interaction parameter $\Lambda = 0.01\pi$. The results for the frequencies $\omega_n = 0.20$, 0.19, and 0.18 are presented row-wise. The many-body results are computed using the MCTDHB method with $M = 6$ time-adaptive orbitals. The convergence is verified with $M = 10$ time-adaptive orbitals. See the text for more details. The quantities shown are dimensionless. Color codes are explained in each panel.

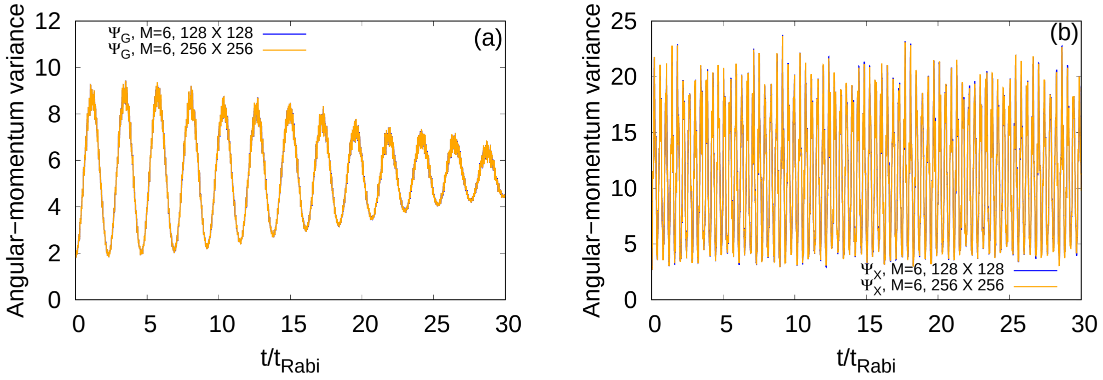

FIG. S15. Convergences of the time-dependent many-body angular-momentum variance of z- component per particle, $\frac{1}{N}\Delta^2_{\hat{L}_Z}(t)$, with the he number of grid points. Initial states are (a) Ψ_G ($\omega_n = 0.19$) and (b) Ψ_X ($\omega_n = 0.18$) in transversely-asymmetric 2D double-well potential. The number of interacting bosons is $N = 10$ and the interaction parameter $\Lambda = 0.01\pi$. The many-body results are computed using the MCTDHB method. The convergence is demonstrated with 128×128 and 256×256 grid points. See the text for more details. The quantities shown are dimensionless. Color codes are explained in each panel.

[1] Streltsov A I, Alon O E and Cederbaum L S 2007 Role of excited states in the splitting of a trapped interacting Bose-Einstein condensate by a time-dependent barrier Phys. Rev. Lett. **99** 030402

[2] Alon O E, Streltsov A I and Cederbaum L S 2008 Multiconfigurational time-dependent Hartree method for bosons: many-body dynamics of bosonic systems Phys. Rev. A **77** 033613

[3] Lode A U J, Lévêque C, Madsen L B, Streltsov A I and Alon O E 2020 Colloquium: Multiconfigurational time-dependent Hartree approaches for indistinguishable particles Rev. Mod. Phys. **92** 011001

[4] Streltsov A I and Streltsova O I MCTDHB-Lab, Version 1. 5. 2015. Available online: (http://mctdhb-lab.com) (accessed on 29 September 2019).

[5] Streltsov A I, Cederbaum L S, Alon O E, Sakmann K, Lode A U J, Grond J, Streltsova O I, Klaiman S and Beinke R The Multiconfigurational Time-Dependent Hartree for Bosons Package, Version 3.x. Available online: http://mctdhb.org (accessed on 29 September 2019).

[6] Klaiman S, and Alon O E 2015 Variance as a sensitive probe of correlations Phys. Rev. A **91** 063613

[7] Klaiman S, Streltsov A I and Alon O E 2016 Uncertainty product of an out-of-equilibrium many-particle system Phys. Rev. A **93** 023605

[8] Alon O E 2019 Analysis of a Trapped Bose-Einstein Condensate in Terms of Position, Momentum, and Angular-Momentum Variance Symmetry **11** 1344

[9] Sakmann K and Schmiedmayer J Conserving symmetries in Bose-Einstein condensate dynamics requires many-body theory arXiv2018, arXiv:1802.03746v2.

[10] Bhowmik A, Haldar S K and Alon O E 2020 Impact of the transverse direction on the many-body tunneling dynamics in a two-dimensional bosonic Josephson junction Sci Rep 10 21476
